# Recent advancements in enzyme-mediated crosslinkable hydrogels: *In vivo*-mimicking strategies

**DOI:** 10.1063/5.0037793

**Published:** 2021-04-01

**Authors:** Wonmoon Song, Junghyeon Ko, Young Hwan Choi, Nathaniel S. Hwang

**Affiliations:** 1 School of Chemical and Biological Engineering, Institute of Chemical Processes, Seoul National University, Seoul 08826, Republic of Korea; 2 Bio-MAX/N-Bio Institute, Institute of BioEngineering, Seoul National University, Seoul 08826, Republic of Korea

## Abstract

Enzymes play a central role in fundamental biological processes and have been traditionally used to trigger various processes. In recent years, enzymes have been used to tune biomaterial responses and modify the chemical structures at desired sites. These chemical modifications have allowed the fabrication of various hydrogels for tissue engineering and therapeutic applications. This review provides a comprehensive overview of recent advancements in the use of enzymes for hydrogel fabrication. Strategies to enhance the enzyme function and improve biocompatibility are described. In addition, we describe future opportunities and challenges for the production of enzyme-mediated crosslinkable hydrogels.

## I. INTRODUCTION

Hydrogels are hydrophilic polymer network structures with high water content. In recent years, hydrogels have been considered one of the most attractive materials for biomedical applications as they can be used to recapitulate tissue-like 3D microenvironments. The diverse and convenient modulation capacity through numerous chemical modifications is also considered an advantage of hydrogels as biomaterial scaffolds. Hydrogel properties depend on the cross-linking mechanisms. There are two main cross-linking mechanisms: physical and chemical cross-linking. Physical cross-linking makes 3D networks by reversible bonding formation, such as van der Waals interactions, crystallization, and hydrogen bonds. In contrast, in chemical cross-linking, irreversible covalent bonds are formed between polymers through chemical agents. Chemically crosslinked networks allow stable hydrogel constructs.

The extracellular matrix (ECM) cross-linking and stability of our tissue structures depend heavily on enzyme-mediated responses. For example, lysyl oxidase mediates collagen or elastin assembly by inducing aldehyde formation from lysine residues of collagen or elastin fibers. Transglutaminase mediates protein cross-linking by the acyl-transfer reaction between the carboxamide group of the glutamate residue and the з-amino group of the lysyl residue. The fabrication of hydrogels using enzyme-mediated cross-linking has been actively studied and shows promising perspectives.^1^ Also, several enzymes active at physiological pH and temperature conditions have been discovered and developed to recapitulate ECMs of living organisms.^2–5^

In addition to the cross-linking activity, enzymes have some unique advantageous properties for hydrogel fabrication. Enzymes catalyze recognizing 3D substrate structures, which fit enzymes for binding. The “substrate specificity” makes tight regulation of enzyme activity without unwanted side reactions.

Despite these prospective advantages of enzymatic cross-linking for biomedical applications, some limitations remain unsolved. Several reaction conditions that may affect the catalytic activity of enzymes significantly, such as pH, temperature, and steric hindrance caused by the substrate structure, make enzymatic cross-linking difficult to use.^6–8^ Moreover, it is difficult to produce recombinant enzymes on a large scale because the size of the gene coding for the recombinant enzyme is too large.^9^ Hence, to solve these problems, protein engineering techniques have been applied to enzymes for cross-linking; for example, the directed evolution process selection of the most compatible enzymes for the function we desire from the initial enzyme by iterative mutant screening. In the protein engineering system, there have been some breakthroughs such as machine learning, which are expected to bring innovations.

The most entrenched method for enzyme screening is the production of recombinant enzymes, that is, a modification of the genetic information that codes for the enzyme.^10–18^ This method is based on the directed evolution, which was developed by Professor George Smith, the 2018 Nobel Prize in chemistry winner. By creating mutant libraries for the initial protein and applying selective pressure, the most compatible enzyme is screened. The approach can be divided into four categories: random mutagenesis, focused mutagenesis, homologous recombination, and circular permutation. Briefly, random mutagenesis is a method of error-prone PCR with a low-fidelity polymerase, and focused mutagenesis replaces a target DNA site with a mutagenic oligonucleotide cassette. Homologous recombination is a method inducing a hybrid of DNA fragments, which causes mutation. Finally, circular permutation attaches the C-term and N-term as linkers and randomizes the new C-term and N-term, dramatically changing the structure of the protein.

There are two methods to produce modified proteins by changing the translation system rather than the genome.^19^ One is called selective-pressure incorporation (SPI), and the other is the stop codon suppression of noncanonical amino acids (SCS). SPI substitutes modified amino acids as the corresponding tRNA cannot differentiate the modification. This process allows the translation of protein-containing modified amino acids. In contrast, SCS focuses on modifying the aminoacyl-tRNA synthetase (AARS) and tRNA (called tRNA_sup_) so that the AARS and its tRNA_sup_ become orthogonal pairs. Nonnatural amino acids can be loaded on tRNA_sup_ that can recognize a stop codon as a normal codon. Through SCS, it is possible to attach nonnatural amino acids with large structures that are difficult to be applied through SPI.

Here, we review the recent advancements in the application of enzymes for hydrogel fabrication. Also, we review recent technological advancements to improve the biological function of crosslinkable enzymes. Finally, hydrogels made by enzymatic polymerization for various applications and future perspectives based on those techniques are discussed.

## II. ENZYMES FOR CROSSLINKING

For hydrogel fabrication, it is important to understand the reaction mechanism to control the reaction rate and conditions with appropriate adjustments and modifications. Regarding the reaction catalyzed by enzymes, an oxidation reaction catalyzed by tyrosinase or peroxidase is one of the main enzyme-mediated cross-linking methods. These enzymes oxidize substrates to reactive forms, which have the potential to make covalent bonds. Also, an acyl-transfer by transglutaminase or a transpeptidation by sortase, which makes bonds between specific amino acids, are examples of enzyme-mediated reactions used in hydrogel fabrication. In this section, we describe the types and mechanisms of enzyme-mediated hydrogel cross-linking and present the modifications to use these enzymes.

### A. Tyrosinase

**FIG. 1.**
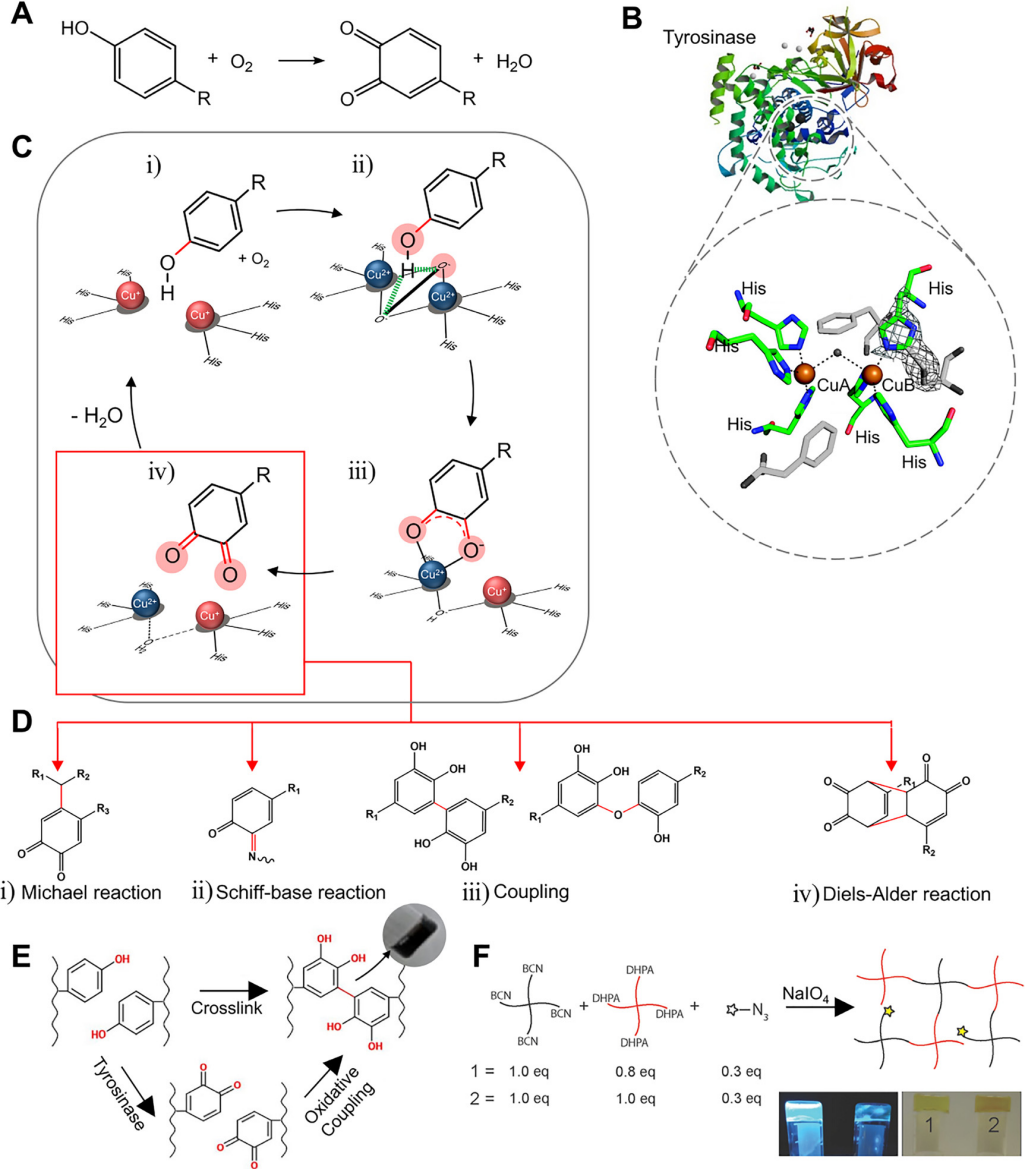
Structure, mechanism, and applications to hydrogel of tyrosinase. (a) Phenol oxidation by tyrosinase. (b) Representative structure of tyrosinase (*A. bisporus*) and its active site. Reprinted with permission from Ismaya *et al.*, Biochemistry **50**, 5477 (2011). Copyright 2011 American Chemical Society.^24^ (c) Schematic illustration of the phenol/catechol oxidation mechanism mediated by tyrosinase. (d) Possible cross-linking mechanisms and products by quinone. Reprinted with permission from Chen *et al.*, Biomaterials **24**, 2832 (2003). Copyright 2003 Elsevier.^3^ (e) Tyramine-modified hyaluronan hydrogel fabricated by tyrosinase. Reprinted with permission from Kim *et al.* Biomaterials **178**, 401 (2018); Copyright 2018 Elsevier.^37^ (f) Hydrogel based on four-armed poly(ethylene)glycol (PEG) with cyclooctyne derivative bicyclo[6.1.0]non-4-yne (BCN) and 3,4-dihydroxyphenylacetic acid (DHPA) fabricated by quinone formation. Reprinted with permission from Jonker *et al.*, Adv. Mater. **27**, 1235 (2015). Copyright 2015 Authors licensed under a Creative Commons Attribution (CC BY) license.^39^

Tyrosinase is an enzyme involved in melanin synthesis, the browning reaction of fruits and vegetables (Maillard reaction), and hardening and darkening of insect cuticles.^20–22^ This enzyme is a copper-containing polyphenol oxidase that oxidizes phenol groups to quinones in the presence of oxygen and without a cofactor [Figs. 1(a) and 1(b)].^23,24^ The mechanism of the phenol oxidation may be understood by analyzing the redox centers. The deoxy-form [Cu(I)-Cu(I)] with two cuprous ions binds to dioxygen to form the oxy-form [Cu(II)-O_2_^2-^-Cu(II)]. The hydroxyl group of the substrate binds to the oxy-form of copper [Fig. 1(c-i-ii)]. Although the detailed structure is controversial, it is known that the deprotonation of the *ortho-*carbon and the reduction of the copper ion lead to the met-form [Cu(II)-Cu(II)] of the redox center, producing a semiquinone [Fig. 1(c-iii)]. The electron transfer from the semiquinone to copper and the proton movement to the oxygen orbital produce the deoxy-form, finally resulting in a quinone [Fig. 1(c-iv].^25,26^

Quinones have high electrophilicity and participate in a variety of reactions such as the Michael and Schiff base reactions, coupling reactions, and cycloaddition reactions. The quinone participating in the Michael reaction is used as an electrophilic acceptor to form C–C bonds with the receptor [Fig. 1(d-i)]. In the case of the Schiff base reaction, the primary amine group attacks the quinone C = O bond, forming a C = N bond [Fig. 1(d-ii)].^27,28^ Additionally, carbon rings in the quinone and catechol groups participate in coupling reactions, which also play an important role in the cross-linking of the hydrogel [Fig. 1(d-iii)].^29–31^ 1,2-Benzoquinone is structurally different from *para-* and *meta-*analogs, allowing various types of reactions. Specifically, in hydrogel cross-linking, the cycloaddition reaction proceeds through the Diels-Alder reaction, a chemical reaction between a conjugated diene and a substituted alkene [Fig. 1(d-iv)]. The *o-*quinone may take part in different modes, using different reactive sites in the ring (carbodiene, heterodiene, carbodienophile, and heterodienophile). A dienophile (uses 3 and 4 carbons as reactive sites) substance reacts with a carbodiene (uses 3, 4, 5, and 6 carbons as reactive sites) to form a bicyclic molecule or reacts with an exposed quinone to form benzodioxin derivatives.^32^ In addition, noncovalent bonding, such as π–π stacking or hydrogen bonding electrostatic attraction, may help to form and maintain matrices. Hence, the selection of a polymer containing phenol residues is an important aspect to consider. For example, casein, gelatin, and fibroin were used as substrates to form hydrogels with tyrosinase without any functionalization.^3,33–35^ Since gelatin has tyrosine residues, Chen *et al.* used gelatin for tyrosinase substrates.^3^ Tyrosinase catalyzes the oxidation of a phenol, forming a quinone. The activated quinone reacts with a primary amine (-NH_2_) of chitosan, proceeding through the Schiff base reaction. Since there are small amounts of tyrosine residues in gelatin, the presence of chitosan is important. The overall blend ratio also needs to be well adjusted. However, it is not enough to have desired mechanical and thermodynamic properties in these cases. Studies have been conducted to attach phenol groups to desired materials. Phenol containing molecules such as tyramine, tyrosine, epigallocatechin gallate (EGCG), catechol, and 3,4-dihydroxyphenylacetic acid (DHPA) attached to backbone materials have been used.^36,185^ Kim *et al.* fabricated tyramine-modified hyaluronan hydrogels with tyrosinase [Fig. 1(e)].^37^ Gelatin was used to provide collagenous microenvironments and hyaluronic acid to contribute to cell proliferation, migration, and tissue regeneration. A tyramine residue was attached to hyaluronic acid to be used as a tyrosinase substrate. Activated quinone residues react with amine, thiol, and imidazole groups of gelatin or other oxidized residues of hyaluronic acid, proceeding through the Michael reaction, Schiff base reaction, and coupling reaction.

Tyrosinase catalyzes the hydroxylation of mono-phenol and the subsequent two-electron oxidation of the resulting catechol to produce the activated quinone. Then, activated quinones react with compounds containing residues such as amines, thiols, imidazole rings, and other phenolic groups to form covalent bonds.^38^ Alternatively, cycloaddition reactions induced using cyclooctyne derivatives can also help cross-linking.^39^ Jonker *et al.* functionalized four-armed poly(ethylene)glycol (PEG) with a cyclooctyne derivative bicyclo[6.1.0]non-4-yne (BCN) and with the 3,4-dihydroxyphenylalanine (DOPA) derivative DHPA [Fig. 1(f)].^39^ BCN, which is a dienophile, attacks the quinone group made by oxidizing DHPA. As in the Diels–Adler reaction, the cycloaddition reaction proceeds to the hydrogel fabrication.

The remaining catechol groups that were oxidized to quinones may be used to help to cross-link. For those remaining groups to participate in gelation, metal ions such as Fe^3+^, Al^3+^, and Ti^4+^ may be added.^40^ The added metal ions and catechol form a ligand complexation producing metal ion coordination bonds, and additionally, metal ions participate in cation-π interaction. Metal ion-catechol coordination bonds are stronger than hydrogen bonds, and cation-π interactions are stronger than π–π interactions. These bonds may participate in stronger cross-linking than other noncovalent bonds.^41^ As briefly mentioned above, not all phenols are oxidized to quinone by tyrosinase. During the oxidation process, catechol and radicals are formed, which also participate in coupling and cross-linking. The details of the reaction are presented in Sec. II B. Choi *et al.* used Fe^3+^ ions so that the remaining oxidized DOPA molecules may interact with Fe^3+^ ions through metal-ligand complexation.^40^ Gelatin was modified with DOPA to provide more substrates for tyrosinase. Some of the oxidized DOPA participates in tissue adhesion and some others in coupling to maintain hydrogel matrices. Fe^3+^ ions help to maintain the hydrogel structure with coordination bonds.

Tyrosinase has the advantage of using various types of materials because it can oxidize most substrates with monophenol residues. In addition, catechol and quinone groups made by oxidation are reactive and may participate in hydrogel formation using various kinds of reactions. These hydrogels show adhesiveness to materials with –SH, phenol, and N=NH groups such as those in skin tissues. Catechol and quinone groups, which did not participate in cross-linking, may participate in the aforementioned adhesive reactions. Also, Huisgen cycloaddition, phosphine-mediated reactions, and carbon nucleophile-mediated cycloadditions, in which reaction quinone participates, have not been studied in the hydrogel field yet. Therefore, these tyrosinases have the potential to produce a variety of hydrogels through various reactions.

### B. Transglutaminase

Transglutaminases, known as biological glues, are Ca^2+^-dependent enzymes that stabilize fibrin clots, elongate fibrin pollen tubes in plants, or cross-link proteins in cell division.^42^ They are also found in plasma, tissues, keratinocytes, and epidermal cells in the human body. Transglutaminases catalyze the formation of an isopeptide bond between the amide group of a glutamine residue and the primary amine group of a lysine residue.^43^

**FIG. 2.**
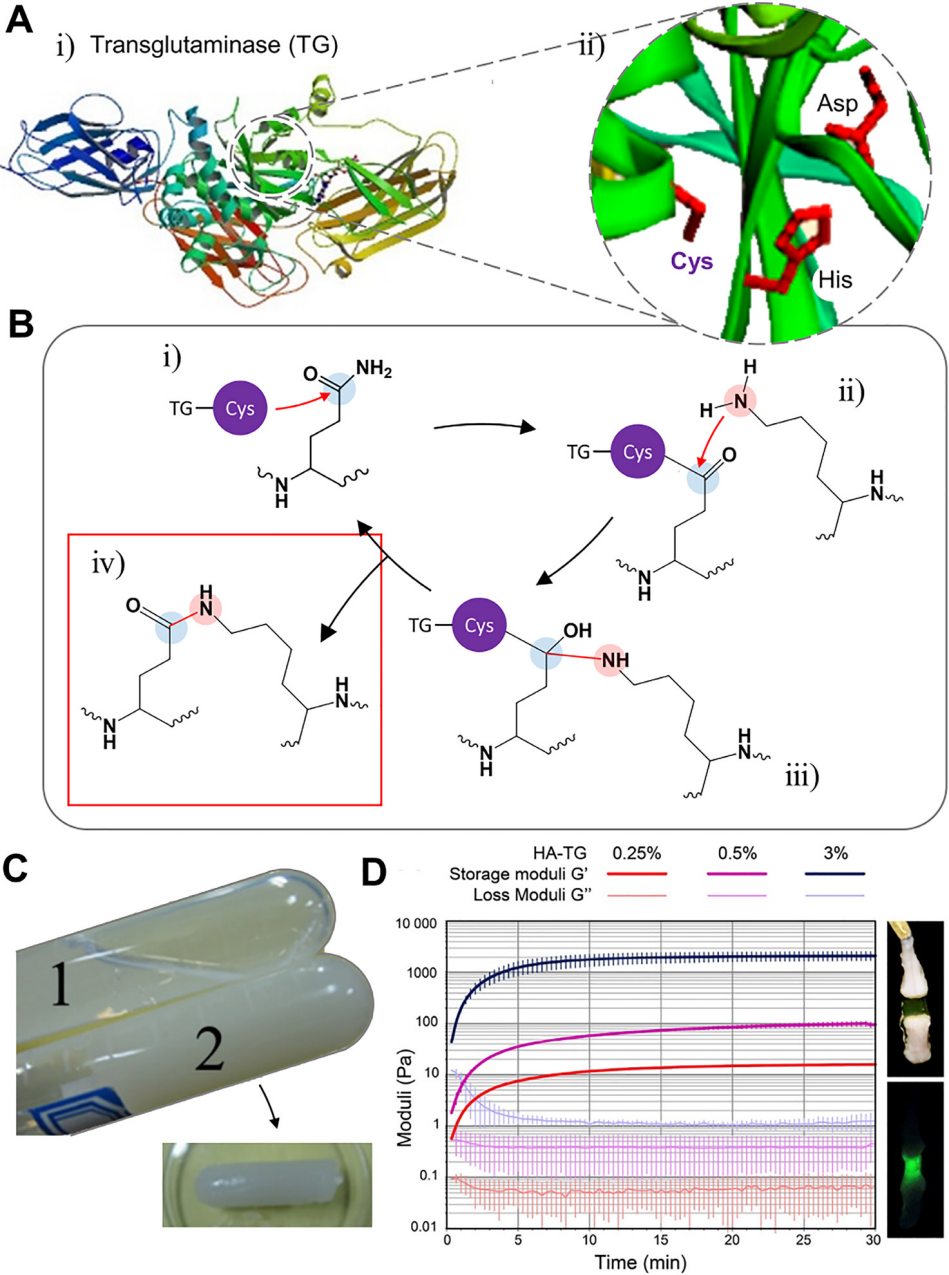
Structure, mechanism, and applications to hydrogel of transglutaminase (TG). (A) Representative structure of transglutaminase [human TG2; (i)] and its active site (ii). Reprinted with permission from Jang *et al.*, PLoS One **9**, e107005 (2014). Copyright 2014 Authors licensed under a Creative Commons Attribution (CC BY) license (i).^44^ Reprinted with permission from Savoca *et al.*, Micromachines **9**, 562 (2018). Copyright 2018 Authors licensed under a Creative Commons Attribution (CC BY) license (ii).^45^ (b) Schematic illustration of the acyl-transfer mechanism mediated by transglutaminase. (c) Hydrogel fabricated by TG, which cross linked human-like collagens. Reprinted with permission from Zhao *et al.*, Mater. Sci. Eng.: C **68**, 317 (2016). Copyright 2016 Elsevier.^75^ (d) Hydrogel fabricated by TG FXIIIa, which cross linked hyaluronan conjugated with glutamine and lysine residues (right) and its rheological properties (left). Reprinted with permission from Broguiere *et al.*, Biomaterials 99, **47** (2016). Copyright 2016 Elsevier.^73^

Active sites are shown in Fig. 2(a), which proceed to the acyl transfer reaction.^44,45^ When the amide portion of the glutamine residue attacks the enzyme as an acyl donor substrate, a thioester intermediate is formed, which leads to an acyl-enzyme form [Fig. 2(b-i)]. In the presence of an acyl-acceptor substrate, such as a lysine residue [Fig. 2(b-ii)], the imidazole group in the active site deprotonates the amine group, while the acyl-enzyme and lysine residues form a tetrahedral group [Fig. 2(b-iii)]. Finally, this group decomposes to create the isopeptide group, forming a crosslinked system [Fig. 2(b-iv)].^46–48^

Various synthetic peptides or biomacromolecules with glutamine and lysine were tested to fabricate hydrogel with the transglutaminase.^49–51,186^ For example, in some cases, the cross-linking between each other is achieved with the enzyme using collagen or gelatin, whey protein, casein, and soy protein, which have both residues at the same time.^3,49,52–71^ Alternatively, cross-linking is performed by adding additional lysine and glutamine residues to a PEG or hyaluronic acid backbone.^72–74^ Zhao *et al.* crosslinked human-like collagen with microbial transglutaminase, fabricating injectable hydrogel [Fig. 2(c)].^75^ Human-like collagen has lysine and glutamine residues at the same time. Therefore, the microbial transglutaminase can catalyze the acyl-transfer, forming inter- or intra-isopeptide bonds. Broguiere *et al.* fabricated transglutaminase FXIIIa crosslinked hyaluronan-based hydrogels, supporting the formation of 3D neuronal networks [Fig. 2(d)].^73^ Hyaluronan, which is the backbone of the ECM in the brain and spinal cord, was functionalized with glutamine and lysine residues. The injectable and tunable hyaluronan-transglutaminase hydrogel was fabricated adding blood coagulation factor XIII. The specific recognition of the FXIIIa substrate amino acid residues helps lowering toxicity during cross-linking.

Transglutaminase does not react with other functional groups, besides those in glutamine and lysine, with high specificity. However, due to this characteristic, the materials that can be used are limited, and therefore, the properties that can be adjusted are also limited, making it difficult to apply to diverse biomedical fields.

### C. Peroxidase

**FIG. 3.**
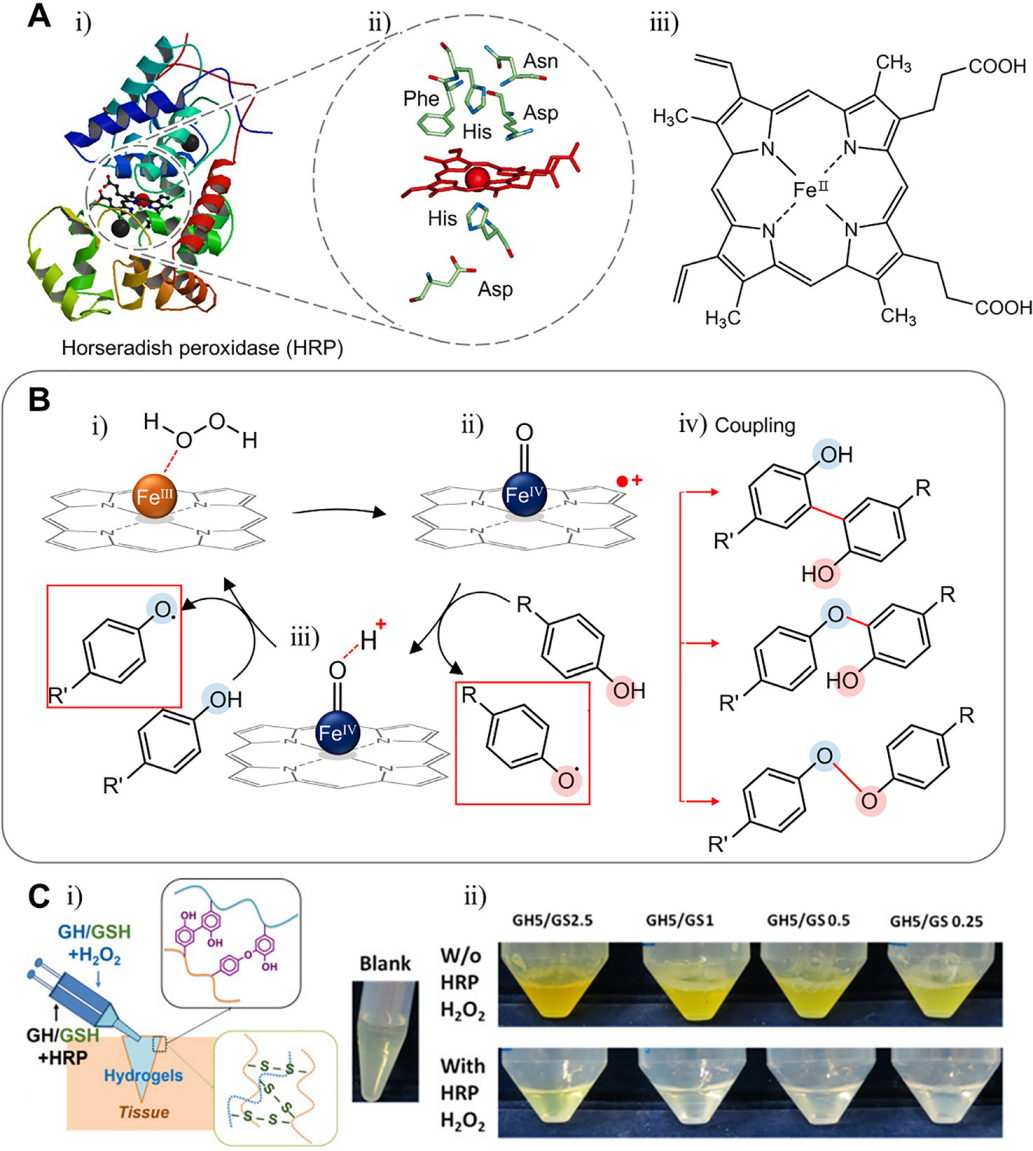
Structure, mechanism, and applications to hydrogel of horseradish peroxidase (HRP). (a) Representative structure of HRP (HRP C1A compound I)^78^ and its active site. Reprinted with permission from Veitch, Phytochemistry **65**, 249 (2004); Copyright 2004 Elsevier. (b) Schematic illustration of the phenol oxidation mechanism mediated by horseradish peroxidase and coupling reaction of radicals. (c) Gelatin-phenol hydrogel cross linked by HRP and H_2_O_2_. Reprinted with permission from Hoang *et al.*, Biopolymers **109**, e23077 (2018). Copyright 2018 Authors licensed under a Creative Commons Attribution (CC BY) license.^92^

The H_2_O_2_ concentration is used as an inter-intracellular signaling molecule, but when the H_2_O_2_ concentration is over 20–50 *μ*M within the cell, cytotoxicity is found in animals, plants, and bacteria.^76^ Peroxidase is found in many organisms and controls the amount of oxidative protection or signaling messenger.^77^ For example, class I enzymes such as ascorbate peroxidases reduce intracellular hydrogen peroxide.^78^ Peroxidase catalyzes the following reaction: H_2_O_2_ + 2 H^+^ + 2 e^-^ → 2 H_2_O. Most of these enzymes use hydrogen peroxide as a substrate, but others use either hydrogen peroxide or lipid peroxide. Several types of peroxidases have been used for immobilization, biosensing, and cross-linking and have been reported to have the potential for hydrogel formation. However, the most used and mimicked peroxidase to produce hydrogels is horseradish peroxidase (HRP). The reason is that HRP has a highly accessible active site and low specificity, and thus, various compounds may be used. The metal center including the heme group in HRP plays an important role in maintaining the structural and functional integrity of the enzyme [Fig. 3(a)].^78^ Mainly, HRP is used to cross-link phenol group-dependent substances with H_2_O_2_ [Fig. 3(b-i)]. While reducing peroxide, the enzyme is oxidized, turning Fe(III) resting states of the heme group to the oxidized state, Fe(IV) oxoferryl center, and porphyrin-based cation radical [Fig. 3(b-ii)]. The compound oxidizes phenol to radicals and becomes an oxoferryl center species [Fig. 3(b-iii)]. Since this compound is also a strong oxidant, it may oxidize phenol and return to the original resting state. The radical produced participates in cross-linking with other phenols or with other radicals [Fig. 3(b-iv)].^79,80^ Therefore, it is very important to have a phenol group in a cross-linking system using HRP. To attach the phenol group, polymers such as dextran, hyaluronan, chitosan, gelatin, PVA, alginate, and carboxymethyl cellulose are used with tyramine and 3-(p-hydroxyphenyl) propionic acid (pHP), among others.^80–90^ Depending on the situation to be used, a hydrogel may be made using the material as gelatin or silk.^91^ Thi *et al.* fabricated tissue adhesive controllable gelatin-based hydrogel [Fig. 3(c)].^92^ Gelatin, which is a well-known polymer because of the high biocompatibility and low toxicity, was used with the incorporation of phenol groups. Applying H_2_O_2_ and HRP to phenol group-modified gelatin, radicals from oxidized phenol groups participate in cross-linking to form hydrogels. Jin *et al.* used dextran grafted hyaluronic acid to mimic the macromolecular structure of proteoglycans.^81^ As an HRP substrate, the tyramine residue was conjugated to dextran. Using N-ethyl-N′-(3-dimethylaminopropyl) carbodiimide hydrochloride (EDAC)/N-hydroxysuccinimide (NHS), the activation reaction of tyramine conjugated dextran is grafted to hyaluronic acid. By controlling the degree of tyramine substitution, the swelling ratio and gelation time may be modified.

Some oxidoreductase such as glucose oxidase (GOx) or laccase can be added to facilitate HRP-mediated cross-linking by generating H_2_O_2_ or active mediators.^93–96^ Also, few researchers proposed that GOx alone can fabricate hydrogels.^97^ However, it is difficult for GOx to directly participate in hydrogel cross-linking since the product itself cannot form a matrix structure. Laccase can also be used as a cross-linking enzyme. For example, it can catalyze substrates to form phenol radicals, which participate in hydrogel fabrication.^98,99^ However, since the polymers used in hydrogel fabrication are too large and have high redox potential for laccase, substrates are too large to enter the active sites and make laccase difficult to be used as a cross-linking enzyme.^100^

Peroxidase-mediated hydrogel fabrication shows fast gelation with low substrate specificity due to the highly reactive hydroxyl radical, which is similar to the cross-link by tyrosinase. However, peroxidase-mediated cross-linking is only induced by radicals. On the other hand, tyrosinase oxidizes substrates to catechol and quinone, as well as radical form. It is the reason that additional optimization or modification is necessary for various situations when using peroxidase.

### D. Sortase

**FIG. 4.**
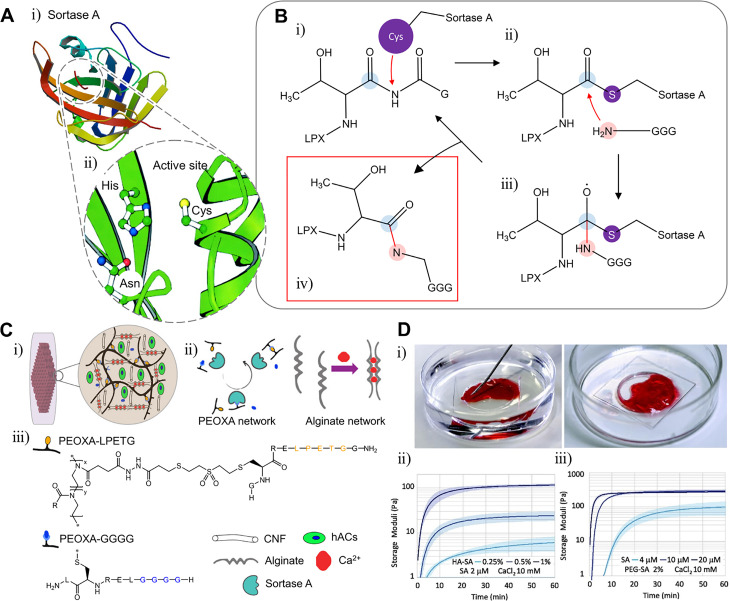
Structure, mechanism, and applications to hydrogel of sortase A. (a) Representative structure of sortase A and its active site. Reprinted with permission from Zong *et al.*, J. Biol. Chem. **279**, 31383 (2004). Copyright 2004 Authors licensed under a Creative Commons Attribution (CC BY) license.^101^ (b) Schematic illustration of the transpeptidation mechanism of sortase A. (c) Hydrogel fabricated by poly(2-ethyl-2-oxazoline) (PEOXA) and alginate (i-ii), which is conjugated with substrates of sortase A, such as LPETG or GGGG (iii). Reprinted with permission from Trachsel *et al.*, Biomacromolecules **20**, 4502 (2019). Copyright 2019 American Chemical Society.^107^ (D) Hydrogel fabricated by sortase A (i), which cross linked the HA-peptide (ii) or the 4-arm-PEG-peptide (iii). Reprinted with permission from Broguiere *et al.*, Acta Biomater. **77**, 182 (2018) Copyright 2018 Authors licensed under a Creative Commons Attribution (CC BY) license.^108^

Sortase is a prokaryotic enzyme present in most gram-positive bacteria [Fig. 4(a)].^101^ This enzyme recognizes the LPXTG (Leu-Pro-any-Thr-Gly) sequence and mediates transpeptidation between threonine and glycine.^102^ By using sortase, the bacteria can display proteins on its surface, scavenge nutrients, adhere to host tissues, invade host cells, and resist host immune responses.

In the cross-linking, the truncated LPXTG portion is attacked by the N-terminal of the GGG-motif to produce a -LPXTGGG- sequence. A cysteine residue of the sortase attacks the carbonyl group of the threonine residue [Fig. 4(b-i)] and forms a sortase-protein complex through a thioacyl bond (vinyl sulfone) [Fig. 4(b-ii)]. Then, the amide group of the glycine residue in the GGG nucleophilically attacks the thioacyl linkage [Fig. 4(b-iii)] to produce an isopeptide [Fig. 4(b-iv)].

To apply sortase to hydrogel fabrication, there have been attempts to attach LPXTG and GGG residues to backbone materials.^103–106^ Trachsel *et al.* fabricated alginate and poly(2-ethyl-2-oxazoline) (PEOXA) based double-network hydrogel for 3D bioprinting [Fig. 4(c-i-ii)].^107^ Ca^2+^ was added to cross-link alginate. To cross-link PEOXA with sortase, PEOXA was conjugated with LPETG and GGGG [Fig. 4(c-iii)]. PEOXA-based single network hydrogel was obtained by adding a sortase solution to a polymer mixture solution. Double–network hydrogel was obtained by adding sortase and CaCl_2_ to a PEOXA mixture and alginate solutions. Furthermore, Broguiere *et al.* crosslinked PEG-based and hyaluronan-based hydrogel with sortase A [Fig. 4(d)].^108^ The 4-arm-PEG-vinylsulfone and HA-vinylsulfone were conjugated with GGGG-LERCL-NH_2_ and GCRE-LPETGG-NH_2_, which are the substrates of sortase A. By mixing equal amounts of peptide-backbone polymers with sortase, gelation was achieved.

## III. PROTEIN/ENZYME ENGINEERING FOR EFFICIENT HYDROGEL FABRICATION

It is well known that the enzyme activity is affected not only by the structure of the substrate but also by pH and temperature. This is the reason why there have been attempts to tailor enzymes to optimal conditions for gelation. However, the factors to be considered for proper enzyme activity are also important for the polymers to produce robust hydrogel networks as well. Therefore, before fabricating a hydrogel, the reaction conditions for enzymes and polymers must be optimized. In this context, various protein-engineering methods have been applied to screen or modify enzymes for enzymatic cross-linking. The enzyme catalytic activity was usually improved, and also, the pH-stability and thermostability were optimized to reaction conditions that are favorable for polymer handling. In this section, we describe previous research on protein engineering techniques that have been applied to enzyme engineering, and thus how the enzyme has been optimized for demanding gelation conditions, and the properties of the resulting hydrogels.

### A. Substrate-specific catalytic activity

The structural relationship between the enzyme and the substrate should be considered as a critical factor in hydrogel fabrication. Substrate specificity is a major characteristic of enzymes, which represents an advantage in enzymatic cross-linking. However, considering that the type of substrate is quite diverse, the substrate specificity of an enzyme is required when it comes to addressing novel monomers. In this context, several studies have been reported on the engineering or screening of novel enzymes.

In fact, in tyrosinase engineering, there have been a few studies on the application to enzymatic cross-linking. Most investigations on tyrosinase engineering focused on catechol production and purification. Catechol may be used for the production of various functional medicines, plastics, antioxidants, and agricultural chemicals. For this reason, tyrosinases were mostly engineered to increase the monophenolase/diphenolase ratio, which generates catechol instead of quinone and hampers the quinone-based cross-linking.^17,109,110^

**FIG. 5.**
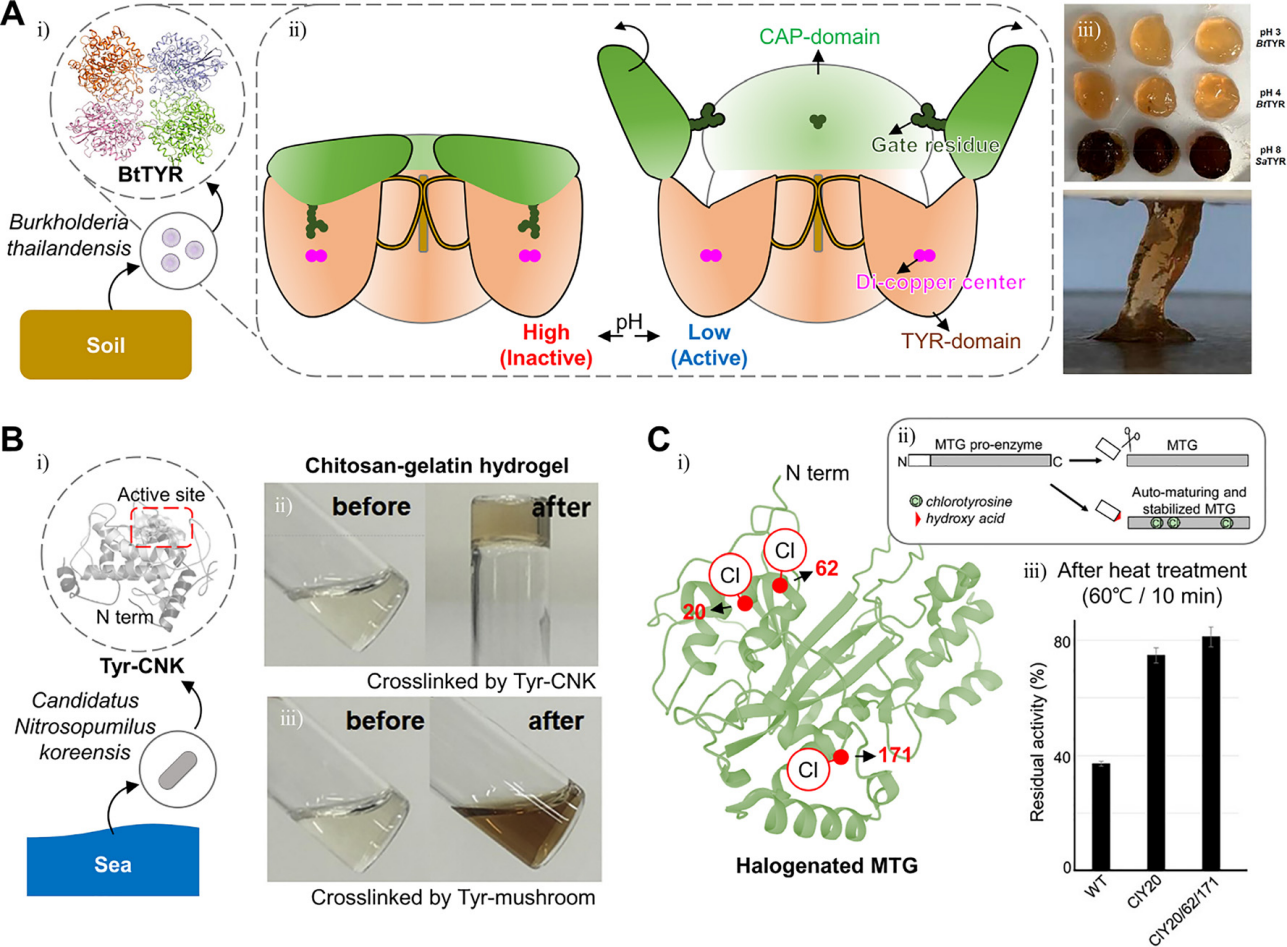
Modified enzymes with enhanced specificity for efficient gelation. (a) Schematic illustration of cyclic permutation (CP) (i) and structure of tyrosinase from *Bacillus megaterium* (*Bm*Ty). The mutation site (CP48) was marked as a red dot (ii). cp48 BmTy showed enhanced catalytic activity on macromolecules (iii). Reprinted with permission from Lee *et al.*, Biomacromolecules **20**, 4502 (2019). Copyright 2019 Authors licensed under a Creative Commons Attribution (CC BY) license.^8^ (b) Schematic of the structural difference between tyrosinase from *Agaricus bisporus* (AB_Ty), *Bacillus megaterium* (BM_Ty), and *Streptomyces avermitilis* (SA_Ty) (i). Comparison of catalytic activity between tyrosinases or substrate types (ii). Adhesive hydrogel fabricated by Bm_Ty (iii). Reprinted with permission from Kim *et al.*, Biomaterials **178**, 401 (2018). Copyright 2018 Elsevier.^37^ (c) Schematic illustration of the gelation mechanism by the protease WQ9-2 (i) and substrate specificity (ii). Hydrogel fabricated by WQ9-2 (iii). Reprinted with permission from Jiang *et al.*, Nano Lett. **17**, 7447 (2017). Copyright 2017 American Chemical Society.^115^

When tyrosinase is used for enzymatic cross-linking, the main hurdle is the cross-linking of phenol or catechol-conjugated polymers. Tyrosinase usually catalyzes monomer substrates faster than polymer substrates as its active site is too deep to contact with polymer molecules. Lee *et al.* demonstrated that tyrosinase engineering may solve that problem. These researchers ligated the N-term and C-term of tyrosinase (BmTy) from *Bacillus megaterium* and inserted a flexible amino acid loop “Thr-Ser-Gly” randomly, which is known as a circular permutation [Fig. 5(a-i)].^8^ Cleaving the random loop changes the enzyme folding pattern and structural stability. Most enzyme mutants lose their reactivity except for 18 variants. However, the cp48 mutant is noteworthy [Fig. 5(a-ii)]. The tyrosine hydroxylase activity of the cp48 mutant decreases compared to the wild-type enzyme, making the BmTy enzyme closer to a catechol oxidase. The cp48 mutant cannot use low-molecular-weight substrates well, such as L-tyrosine and L-DOPA in contrast to macromolecules such as gelatin and daidzin [Fig. 5(a-iii)].

Kim *et al.* used tyrosinase that is more active with polymer substrates than with monomers.^37^ These authors demonstrated that the tyrosinase from *Streptomyces avermitilis* (SA-Ty) may use polymers better than other tyrosinases due to its exposed active site [Fig. 5(b-i)]. The exposed active site can make SA-Ty use polymers well, with a lower steric hindrance. Also, these authors fabricated a hydrogel with more adhesive, rapid forming capacity using this tyrosinase [Fig. 5(b-ii-iii)].

Sortase A (SrtA) has excellent kinetics and specificity and has been actively studied in protein conjugation. In particular, Chen *et al.* developed a directed evolution platform that increases the efficiency of bond-forming enzyme screening significantly.^12^ The process begins with the fabrication of a yeast display, which expresses a mutant SrtA on the yeast surface. Then, specific substrates for SrtA are treated, resulting in substrate conjugation by SrtAs. Among the yeast library, yeast enzymes that use substrates more efficiently than others were sorted by FACS. As a result, most high-performance bond-forming enzymes are screened. Based on this directed evolution system, a screened SrtA improved its catalytic activity up to 140 times.

There have been several reports on enzymatic cross-linking using engineered SrtAs. Arkenberg *et al.* crosslinked YLPRTG- or GGGG-conjugated 8-Arm PEG with a SrtA heptamutant (P94R, E105K, E108Q, D160N, D165A, K190E, and K196T). By additional treatment of mushroom tyrosinase after gelation, the stiffness of the hydrogel is enhanced.^111^ Similarly, Broguiere *et al.* demonstrated that the hydrogel may be rapidly produced by cross-linking LPXTG-or GGGG-conjugated hyaluronate or 4-Arm PEG with a SrtA pentamutant.^108^ Furthermore, Arkenberg *et al.* also reported that the physical properties of the hydrogel may be effectively controlled by the reaction time of the SrtA heptamutant and soluble glycine.^112^

In contrast, although it is a bond-forming enzyme-like SrtA, transglutaminase (TG) is more difficult to engineer for enzymatic cross-linking. According to Deweid *et al.*, the yeast display-based directed evolution system is not useful for TG as it is for SrtA. Deweid *et al.* suggested that the low specificity of TG that recognizes glutamine and lysine as substrates makes TG engineering difficult.^15^ There have been several reports on TG engineering based on directed-evolution that enhanced the TG substrate specificity and catalytic activity.^15,16,113^ Reports referring to the application of engineered TG to hydrogel fabrication are scarce.

Another perspective to modify the enzyme specificity is the spatiotemporal control based on enzyme-caging techniques, although there have been few reports of hydrogel fabrication based on enzyme caging, due to the difficulties in maintaining the stability and activity of enzymes after bioconjugation.^114^ In some cases, enzymes were modified by caging scaffolds, but this has not been applied to hydrogel fabrication. Jiang *et al.* increased enzyme specificity without any direct modification of the enzyme structure [Fig. 5(c-i)].^115^ The zinc metalloprotease (WQ9–2) was encapsulated within a protein nanocapsule called W-NC. By doing that, the contact between WQ9–2 and large substrate molecules such as hirudin or TNF-related apoptosis-inducing ligand (TRAIL) was inhibited [Fig. 5(c-ii)]. Therefore, only small substrate molecules such as Fmoc-F/FF-Dopa might diffuse into W-NC and polymerize through WQ9–2. As a result, an oligopeptide hydrogel was formed by self-assembly of Fmoc-FFF-Dopa [Fig. 5(c-iii)].

In some cases, enzymes were modified by caging scaffolds, but this has not been applied to hydrogel fabrication. Zhang *et al.* conjugated HRP to the poly(acrylamide-co-acrylonitrile) scaffold, and the activity of the HRP was regulated by the scaffold photothermal phase transition.^116^ After phase transition under the upper critical solution temperature (UCST) condition, enzyme substrates diffuse into the gel so that collision reactions between the enzyme and the substrate occur. Nucleic acid scaffold-based enzyme caging techniques make various platforms for enzyme activity regulation. Enzyme-substrate collision may be regulated by changing the DNA nanocage structures under various stresses such as light, temperature, and specific substrates.^117–119^

### B. Enzyme stability under specific pH or thermal conditions and enzyme biocompatibility

Enzyme activity is strongly affected by the pH and temperature. In enzymatic cross-linking, the hydrogel is usually crosslinked within the physiological pH and temperature range.

**FIG. 6.**
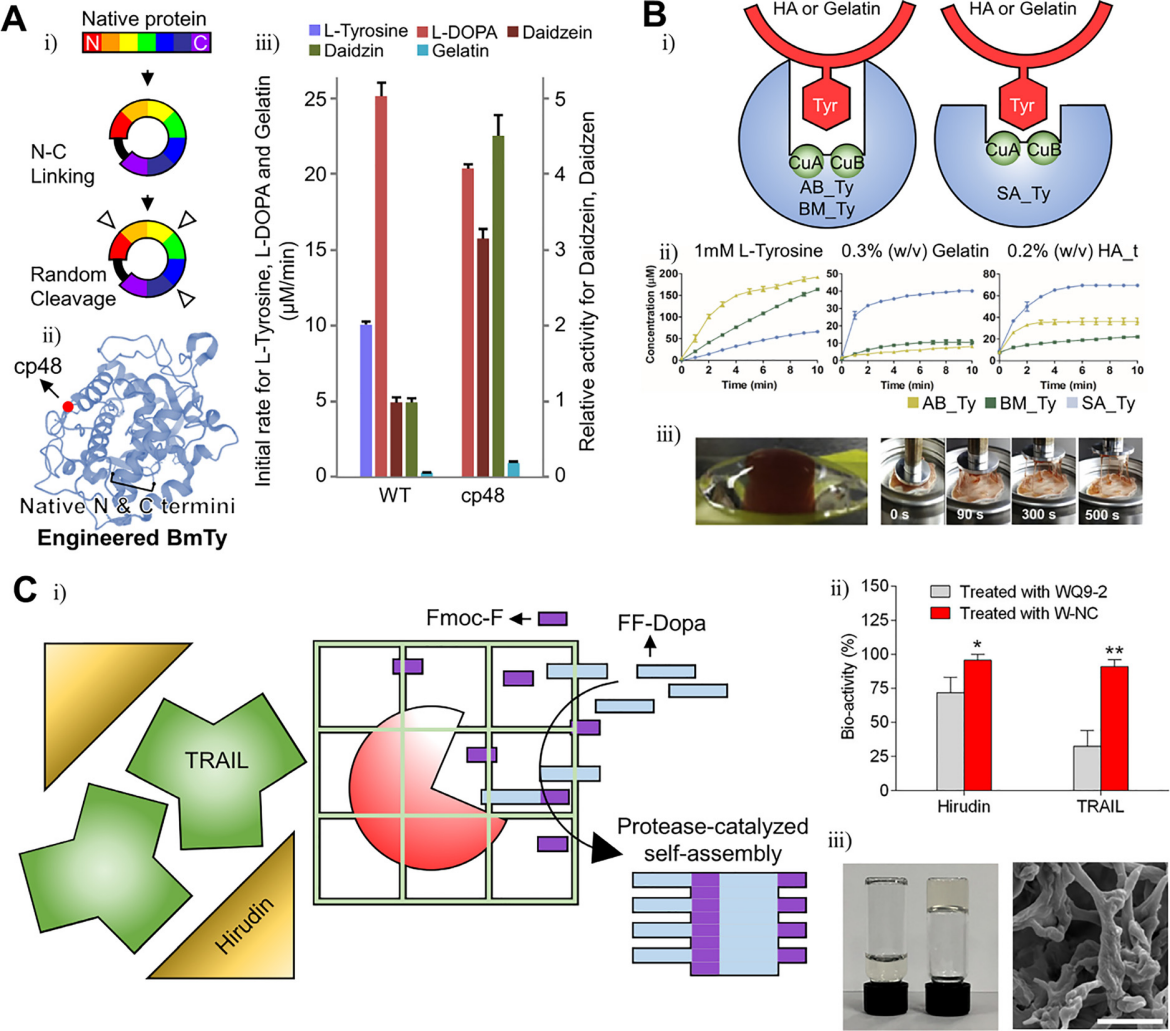
Modified enzymes with enhanced stability for efficient gelation. (a) Schematic illustration of the tetrameric structure of tyrosinase from *Burkholderia thailandensis* (BtTYR) (i) and its pH-dependent shape change (ii). Hydrogel fabricated by BtTYR showed enhanced stability and adhesiveness (iii). Reprinted with permission from Son *et al.*,^120^ ACS Catalysis 8, 10375 (2018); Copyright 2018 American Chemical Society. (B) Schematic illustration of the structure of tyrosinase from *Candidatus Nitrosopumilus koreensis* (Tyr-CNK) (i) and hydrogel fabricated by Tyr-CNK (ii), compared with mushroom tyrosinase (iii). Reprinted with permission from Choi *et al.*, Biochemical Engineering Journal 129, 50 (2018); Copyright 2018 Elsevier.^124^ (C) Structure of halogenated microbial transglutaminase (MTG) (i) and its schematic illustration (ii). Halogenation sites (29, 62, and 171) were marked as red dots. Halogenated MTG showed enhanced thermostability (iii). Reprinted with permission from Ohtake *et al.*, ACS Synth. Biol. **7**, 2170 (2018). Copyright 2018 American Chemical Society.^125^

Son *et al.* discovered a novel tyrosinase, BtTYR, from the soilborne micro-organism *Burkholderia thailandensis* and that it can maintain its catalytic activity under acidic conditions between pH 3 and 5 [Fig. 6(a-i)].^120^ They compared its catalytic activity with catalytic activities of other tyrosinases from *Agaricus bisporus* (AbTYR), *B. megaterium*, and *S. avermitilis* (SaTYR). Son *et al.* demonstrated that the enzyme from *B. thailandensis* (BtTYR) showed eminent DOPA oxidation capacity under acidic conditions (pH 3 and 4) [Fig. 6(a)]. Also, the mechanism that is mediated by its unique tetrameric structure and active site exposure at acidic pH was elucidated [Fig. 6(a-ii)]. When the pH changes from basic to acidic, the inactive BtTYR is activated by opening its CAP domain and exposing its active site. Furthermore, due to the acidic pH, which reduces DOPA oxidation significantly, the BtTYR-mediated DOPA hydrogel cross-linking in acidic pH can make the gel very sticky and long-lasting [Fig. 6(a-iii)]. In contrast, other DOPA hydrogels fabricated through conventional tyrosinases under basic conditions (pH 8) became darker, which means that quinone formation from DOPA occurred by excessive oxidation of DOPA. Also, BtTYR-mediated DOPA hydrogel showed twofold higher stickiness than SaTYR-mediated hydrogel.

Stable hydrogels require not only pH-robust enzymes but also enzymes that are stable at various temperatures. For example, in the case of BtTYR, low temperature prevents DOPA from excessive autooxidation. The halogenation of amino acids in enzymes has been reported to improve thermostability. Halogens such as chloride have bulky and electronegative characteristics, inducing steric expansion and dipole moment change in aliphatic amino acid residues.^121^ Although most enzymes lose their activity after halogenation because of the unstable structure produced by the steric expansion, some specific sites can accommodate these changes. Thermostable enzymes may be obtained through screening.^122^

A stable tyrosinase that can even maintain activity at 0 °C has been reported. Kim *et al.* discovered a novel tyrosinase by searching the tyrosinase genome sequence (NCBI reference sequence, AFS80363) in BLAST [Fig. 6(b-i)].^123^ The tyrosinase genome of an archaeon from deep marine sediments called *Candidatus Nitrosopumilus koreensis* was unveiled, and its tyrosinase is referred to as Tyr-CNK. Do *et al.* conducted further research demonstrating that Tyr-CNK has stable activity at lower temperatures (0–20 °C) than the conventional mushroom tyrosinase.^110^ Furthermore, Choi *et al.* demonstrated that the fabrication of chitosan/gelatin hydrogel by Tyr-CNK-mediated cross-linking is also possible [Fig. 6(b-ii)].^124^

Thermostable enzymes are also useful to handle gelatin-based hydrogels because those protein polymers may become reactive easily if denatured at high temperature. Ohtake *et al.* halogenated several tyrosine residues in TG [Fig. 6(c-i-ii)].^125^ The chlorination of the amino acids 20, 52, and 171 in transglutaminase results in a more stable enzyme with a 5.1-fold longer half-life at 60 °C than that of the wild type enzyme [Fig. 6(c-iii)].

In general, horseradish peroxidase (HRP) has been extracted from the root of *Armoracia rusticana*. The demands for recombinant and engineered HRPs increased because of the inconveniences related to the plant, such as seasonality, difficult cultivation, and low yield. Therefore, several efficient recombinant HRPs were produced using powerful screening methods.^126^ However, those HRPs of plant origin were difficult to use in human medicine because of their immunogenicity. The main cause of HRP immunogenicity is glycosylation. Thus, by adding ER retention sequences to HRP for reducing mannose-type glycosylation, HRP-inducible immunogenicity may be reduced. Furthermore, Humer *et al.* made a nonglycosylated quadruple HRP mutant by modifying the 13, 57, 255, and 268 asparagines in HRP. As a result, the mutant HRP showed twofold longer half-life and eightfold higher activity than the control.

## IV. RECENT APPLICATIONS OF ENZYME-MEDIATED CROSSLINKABLE HYDROGELS IN REGENERATIVE MEDICINE

Enzyme-mediated crosslinkable hydrogels have been used for various strategies in regenerative medicine. The properties of modified hydrogels with functional groups and enzyme-mediated cross-linking mechanisms have facilitated bioprinting, injection/spraying, and tissue adhesion/hemostasis (Table I). In this section, we present several recent applications using enzyme-mediated crosslinkable hydrogels in regenerative medicine.

**TABLE I.** Applications of enzyme-cross-linked hydrogels and their advantages.

Application	Enzyme	Substrate polymer	Advantages	References
Bioprinting	Tyrosinase	Collagen-tyramine	Skin regeneration	130
	Tyrosinase	Silk fibroin, gelatin	Osteogenesis, Chondrogenesis	128
	Transglutaminase	Gelatin methacryloyl	Stable rheological property	129
	Horseradish peroxidase	Chondroitin sulfate-tyrosine	Adjustable mechanical strength	127
	Horseradish peroxidase	Gelatin-tyramine, hyaluronate-tyramine	High cell viability	80
	Sortase	Poly(2-ethyl-2-oxazoline)-LPETG(Peptide), Poly(2-ethyl-2-oxazoline)-GGGG	Fast kinetics, high cell viability	107
Adhesives, hemostats	Tyrosinase	Gelatin-DOPA	Hemostasis on a liver defect	40
			Human-derived gelatin	
	Tyrosinase, horseradish peroxidase	Gelatin-hydroxyphenyl propionic acid	Adjustable gelation time	134
	Horseradish peroxidase	Poly(γ-glutamic acid)-dopamine	Wet adhesion	136
			Hemostasis on a liver defect	
	Horseradish peroxidase	Chitosan-PEG-tyramine	High adhesive strength	137
			Hemostasis on a liver defect	
			Skin regeneration	
	Horseradish peroxidase	Gelatin-hydroxyphenyl propionic acid, gelatin-thiol	Adjustable gelation time	92
			High adhesive strength	
Injection, spraying	Tyrosinase	Hyaluronic acid-tyramine, gelatin	High adhesive strength	37
			High mechanical strength	
			Skin regeneration	
	Transglutaminase	Human-like collagen	Adjustable degradability	75
	Horseradish peroxidase	Gelatin-hydroxyphenyl propionic acid	Loading and release of cytokines	138
			Wound healing	
	Horseradish peroxidase	Gelatin-thiourea−catechol	Adhesiveness	135
			Fast gelation	
Therapeutic agent delivery	Tyrosinase	Chitosan-EGCG	EGCG loading	185
			Antibacterial, antioxidant, anti-inflammatory effect	
	Transglutaminase	Human-like collagen	bFGF loading	56
			Wound healing	
	Transglutaminase	Human-like collagen, fish bone collagen	[EMIM^+^][Ac^-^] loading	57
			Anticancer effect *in vitro*	
	Transglutaminase	Casein, Konjac glucomannan	Docetaxel loading	64
			Cumulative release	
	Transglutaminase	Soy protein isolate	Riboflavin loading	186
			Adjustable release rate	
	Horseradish peroxidase	Silk-tyramine, gelatin-tyramine	Cell encapsulation	139
			Adjustable mechanical strength	
Tissue regeneration	Tyrosinase	Hyaluronate-tyramine	Cartilage regeneration	140
	Transglutaminase	Collagen	Skin regeneration	55
			Thermal stability	
	Transglutaminase	Hyaluronate-NQEQVSPLERCG (Peptides), hyaluronate-FKGGGPQGIWGQERCG	Neural regeneration	73
			Injectability	
	Horseradish peroxidase	PVA-tyramine	Skin regeneration	83
			Fast gelation	

### A. Application to bioprinting

Natural ECM-derived hydrogels are ideal bioprinting materials for applications to tissue engineering and regenerative medicine due to their cytocompatibility and biocompatibility. Also, the enzymatic polymerization provides several advantages for bioprinting such as the proper extrusion ability and printability with the controllable activity of the enzyme and mild reaction conditions compared to those of traditional polymerization.^107,127,128^

**FIG. 7.**
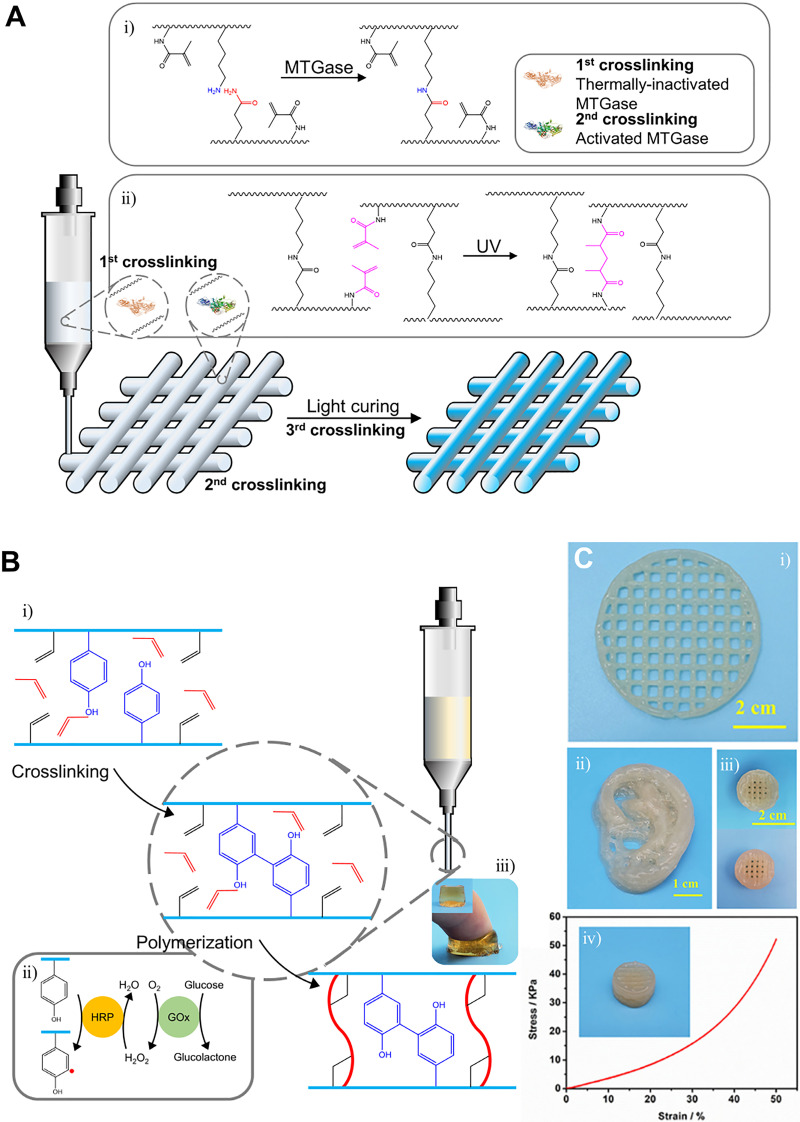
3D printing systems mediated by enzymatic cross-linking. (a) Schematic illustration of the MTGase-mediated, adjustable GelMA bioink system. Reprinted with permission from Zhou *et al.*, Biofabrication **11**(2), 025011 (2019). Copyright 2019 Authors licensed under a Creative Commons Attribution (CC BY) license.^129^ (b) Schematic illustration of printing of biocompatible hydrogel with the dual-enzyme cross-linking system. Preparation of Gel I and Gel II systems (i). Serial reactions with the dual enzyme and formation of α-carbon radicals (ii). Hydrogel product (iii). (c) Dual-enzyme cross-linking systems enable fabrication of complex structures—lattice (i), ear (ii), and button with enhanced mechanical properties (iii-iv). Reprinted with permission from Shen *et al.*, Front. Chem. **8**, ▪ (2020). Copyright 2020 Authors licensed under a Creative Commons Attribution (CC BY) license.^127^

Zhou *et al.* facilitated the control of the rheological property of gelatin methacryloyl (GelMA) hydrogel through modulating the activity of the enzyme [Fig. 7(a)].^129^ Briefly, these researchers introduced Ca^2+^-independent microbial transglutaminase (MTGase) in the GelMA solution to partially catalyze covalent bond formation [Fig. 7(a-i)]. Then, the enzyme was deactivated to keep the hydrogel extrusive and printable. After printing, a post-photo cross-linking step was, then, introduced to enable the long-term stability of the printed structure [Fig. 7(a-ii)]. Similarly, Shi *et al.* adopted a two-step cross-linking system, enzymatic cross-linking, and post-photo cross-linking.^130^ Crosslinking of collagen with mushroom tyrosinase was adopted as the pre-cross-linking step, and photo-cross-linking of GelMA was adopted to form a mechanically stable 3D structure.

Shen *et al.* designed biocompatible and mechanically adjustable bio-ink with dual enzyme systems [Fig. 7(b)].^127^ Tyrosine and glycidyl methacrylate modified chondroitin sulfate (GMA-CS-Ph-OH) were synthesized as biocompatible polymers [Fig. 7(b-i)]. HRP, glucose oxidase (GOx), glucose, and acrylamide monomers were added to the polymer solution. First, GOx oxidized glucose to gluconic acid and H_2_O_2_, and then, HRP and H_2_O_2_ oxidized tyrosine to cross-link CS (Gel I) [Fig. 7(b-ii)]. In addition, oxidized tyrosine formed α-carbon radicals, which can gradually initiate cross-linking between acrylamide monomers and glycidyl methacrylate grafted to CS (Gel II) [Fig. 7(b-iii)]. The Gel I system enabled extrusion and conveniently printing, while the Gel II system enabled modulation of mechanical strength from 3.29 to 86.73 kPa along with various concentrations of monomers [Fig. 7(c)].

### B. Application to tissue adhesion/hemostasis

**FIG. 8.**
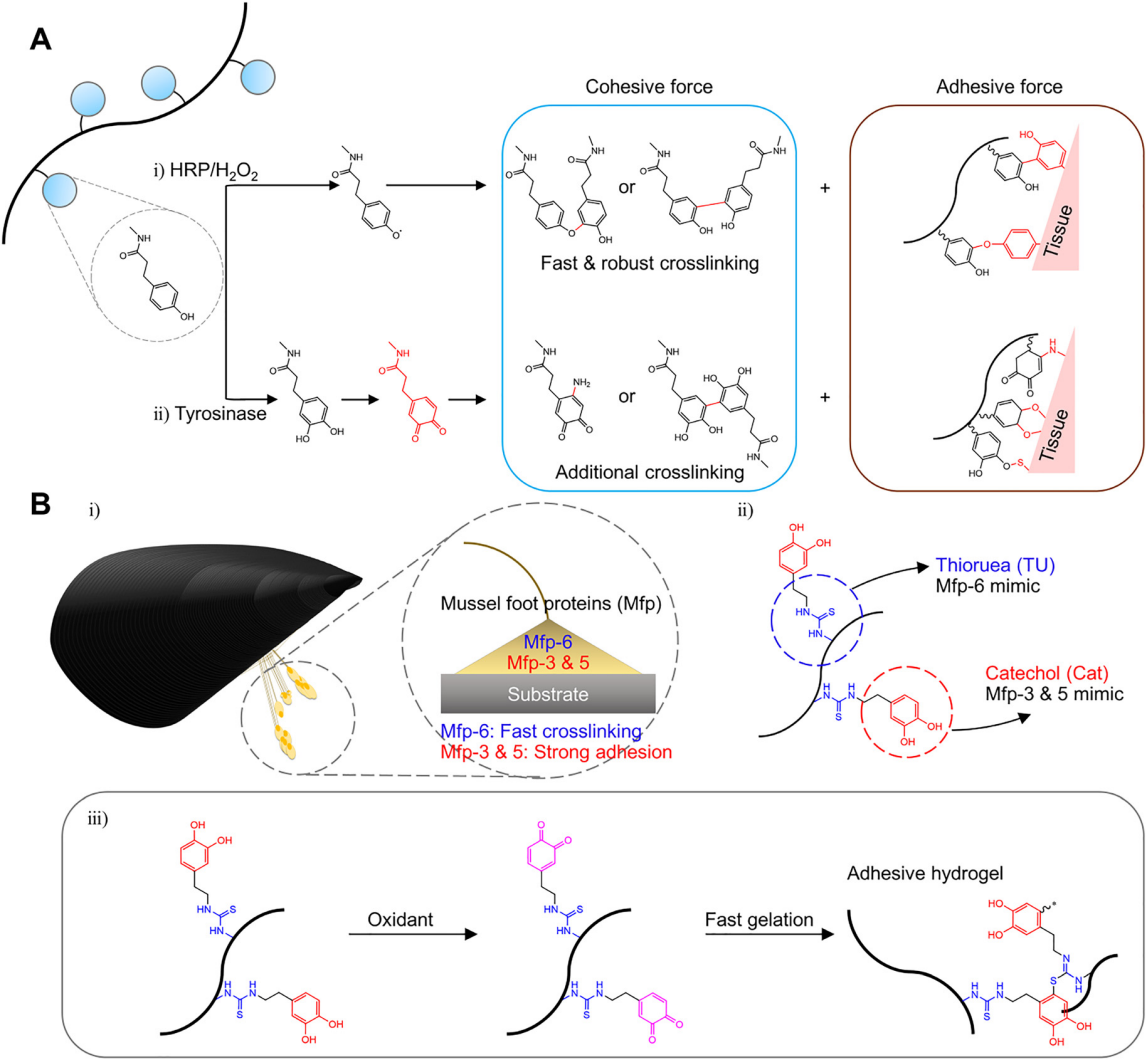
Hydrogels for tissue adhesives mediated by enzymatic cross-linking. (a) Schematic illustration of formation of highly adhesive hydrogels via HRP and the tyrosinase cross-linking system. Republished with permission from Thi *et al.*, J. Mater. Chem. B **5**, 757 (2017). Copyright 2017 Royal Society of Chemistry, conveyed through Clearance Center, Inc. (b) Schematic illustration of the two types of mussel foot protein-inspired injectable gelatin hydrogel-tissue adhesives (i-ii). The cross-linking mechanism of catechol-rich proteins (adhesive property, Mfp-3 and Mfp-5), thiol-rich protein (cross-linking property, Mfp-6), and designed mussel-inspired gelatin hydrogel (iii). Reprinted with permission from Wei *et al.*, ACS Appl. Mater. Interfaces **11**, 51 (2019). Copyright 2019 American Chemical Society.^135^

Tissue adhesive hydrogels have been designed for hemostasis, as sealants, dressing, and minimally invasive therapy.^92,131–133^ Thi *et al.* fabricated enhanced tissue adhesive hydrogel using a dual-enzymatic (HRP and mushroom tyrosinase) cross-linking system [Fig. 8(a)] for tissue regenerative applications.^134^ HRP induced hydrogel-tissue adhesion by forming di-phenol coupling between tyrosine groups of the tissue surface and the phenol groups of gelatin [Fig. 8(a-i)]. In addition, tyrosinase induced additional strong adhesion by converting phenol moieties into highly activated quinones [Fig. 8(a-ii)]. These quinones can react with various nucleophiles such as -NH_2,_ -SH, or -OH and form a covalent bond. Wei *et al.* reported dual-mussel foot proteins (Mfps) mimicking gelatin hydrogels to achieve excellent wet adhesion [Fig. 8(b-i)].^135^ Thiourea-catechol modified gelatin hydrogels, which mimic Mfp-6 and Mfp-3&5, showed an injectable hydrogel adhesive via dual-syringe and HRP cross-linking systems with fast gelation and strong adhesion properties [Fig. 8(b-ii)]. Thiol-rich Mfp-6 is related to not only rapidly cross-linking of the catechol-rich proteins (Mfp-3&5) but also reducing the oxidized quinones back to the catechol group [Fig. 8(b-iii)].

Chen *et al.* designed a hemostatic agent with dopamine modified poly(γ-glutamic acid) hydrogel via an HRP-mediated cross-linking.^136^ They identified that the designed hydrogel showed ten times stronger tissue adhesion and excellent hemostatic activity than that of the fibrin glue. Lih *et al.* prepared chitosan-poly(ethylene glycol)-tyramine (CPT) hydrogels for hemostasis and wound healing application with the rapid cross-linking ability and tissue adhesiveness using HRP and hydrogen peroxide.^137^

### C. Application to injection/spraying

**FIG. 9.**
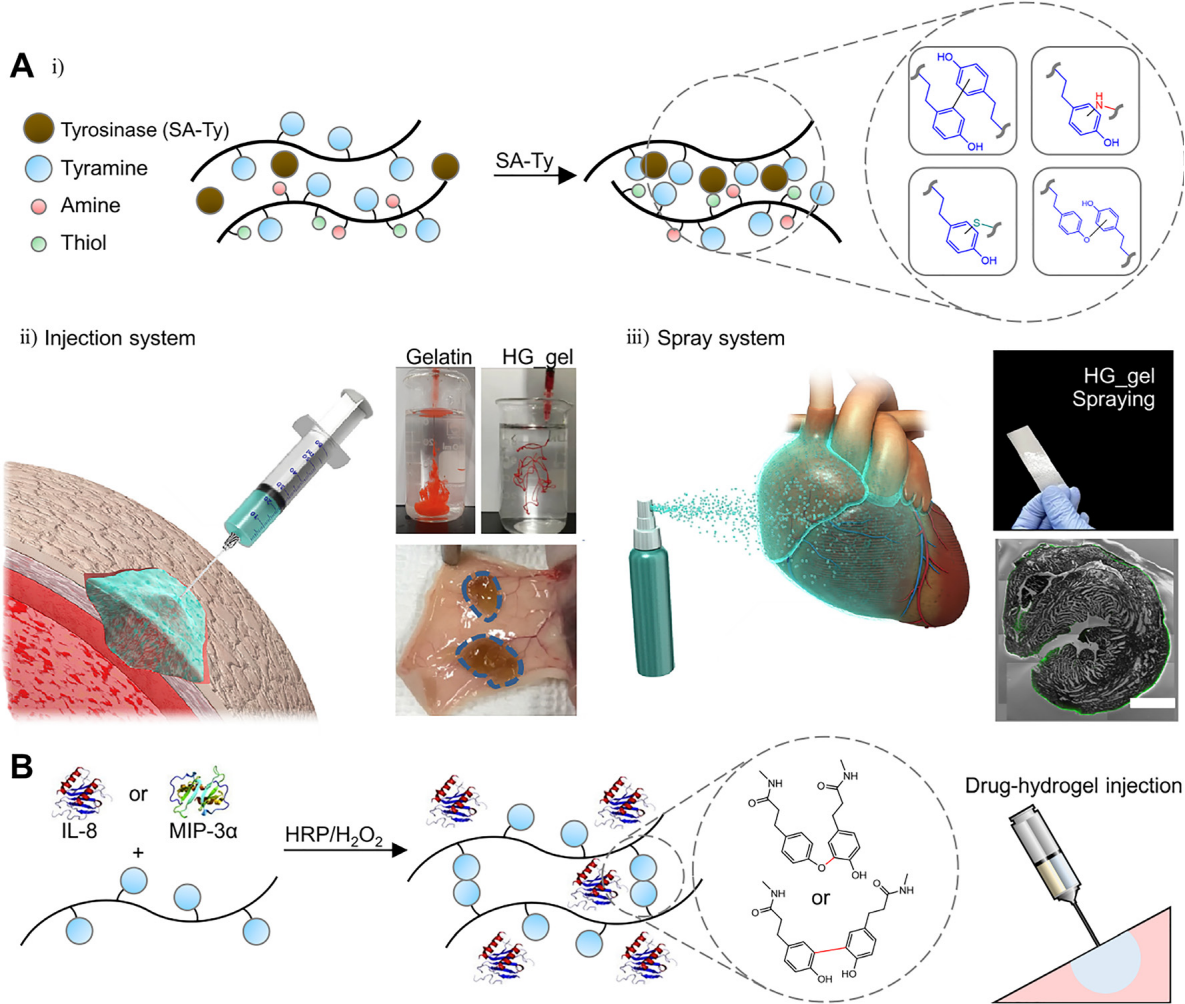
Hydrogels for tissue injection and spraying mediated by enzymatic cross-linking. (a) Schematic illustration of SA_Ty-mediated cross-linking of HG_gel (i). Injectability of HG_gel and *in vivo* injection (ii). Sprayable system of HG_gel via airbrush and *ex vivo* coating of gel on the cardiac tissue (iii). Reprinted with permission from Kim *et al.*, Biomaterials **178**, 401 (2018). Copyright 2018 Elsevier.^37^ (b) Schematic illustration of chemokine-loaded sprayable GH hydrogels for diabetic wound dressing. Reprinted with permission from Yoon *et al.*, Acta Biomater. **38**, 59 (2016). Copyright 2016 Elsevier.^138^

Enzyme-mediated crosslinkable hydrogels with injectable or sprayable properties have been designed with a dual syringe system or a controllable cross-linking system.^75^ Kim *et al.* reported injectable and sprayable ECM hydrogels by optimizing the cross-linking conditions with novel tyrosinase [Fig. 9(a)].^37^ Tyramine-conjugated hyaluronic acid (HA_t) and gelatin hydrogel (HG_gel) were rapidly formed with novel tyrosinase (SA_Ty) derived from *S. avermitilis*, which has superior reactivity compared to previous tyrosinases [Fig. 9(a-i)]. Furthermore, a proper concentration of sodium chloride was added to the medical syringe and commercial spray bottle loading HG and SA_Ty mixture to inhibit undesirable cross-linking before use [Fig. 9(a-ii-iii)]. When the mixture was injected or sprayed into the wet environment such as the surface of the organ or tissue, the rapid cross-linking occurred through dilution of the sodium chloride concentration, which enables the enzyme to function. Through these controllable cross-linking mechanisms and superior reactivity of the novel tyrosinase, these injectable and sprayable systems have shown potential use in tissue engineering and regenerative medicine.

### D. Application to therapeutic agent delivery

Yoon *et al.* developed an injectable gelatin hydrogel using a dual syringe system for diabetic wound dressing [Fig. 9(b)].^138^ Inflammatory cell recruiting chemokines were loaded into gelatin hydrogels modified with hydroxyphenyl propionic acid (GH hydrogels) during the HRP-triggered *in situ* cross-linking reaction. Chemokine-loaded sprayable GH hydrogels have promoted wound regeneration with effective reepithelialization, neovascularization, and collagen deposition in the streptozotocin-induced diabetic mice wound model. Hasturk *et al.* designed tyramine and cyclic arginine-glycine-aspartic acid (RGD)-modified silk fibroin and tyramine-modified gelatin hydrogels for cell encapsulation.^139^ The crosslinking density, mechanical properties, degradation, and bioactivity of cells were tunable through varying the amount of silk fibroin, gelatin hydrogels, and HRP/H_2_O_2_.

### E. Application to tissue regeneration

With nontoxic, highly efficient, stable properties and drug-loading techniques, enzyme cross-linking-based hydrogels have also been applied to research studies of tissue regeneration. Jin *et al.* developed an anti-inflammatory hydrogel for osteoarthritic cartilage repair by cross-linking hyaluronate-tyramine conjugates and gelatin using tyrosinase.^140^ One of the main limitations in current methods for osteoarthritis therapy is the burst release of drugs and fast degradation of scaffolds.^141^ By providing a stable hydrogel system based on hyaluronate and loading anti-inflammatory plant metabolite “epigallocatechin gallate(EGCG),” Jin *et al.* demonstrated that this anti-inflammatory hydrogel is quite effective to alleviated symptoms in the murine osteoarthritis model. Jiang *et al.* developed collagen-fibril hydrogel crosslinked by transglutaminase for skin regeneration.^55^ When 40 U/g of transglutaminase was treated to collagen, transglutaminase efficiently made collagen self-assembled. Jiang *et al.* demonstrated that the thermostability of collagen fibrils could be increased by adjusting the transglutaminase concentration, varying the thermal denaturation temperature from 60.6 (collagen only) to 82.8 °C (with 80 U/g of transglutaminase). This thermostable hydrogel (with 40 U/g of transglutaminase)-treated rat wound models showed significantly fast wound healing rates, about 1.5 times faster than the groups that were treated hydrogel without transglutaminase in 2 weeks.

## V. NEXT-GENERATION PROTEIN ENGINEERING STRATEGIES FOR ENZYME-CROSSLINKABLE HYDROGEL FABRICATION

Despite the rapid progress of enzyme engineering thanks to powerful tools such as directed evolution and computational engineering, the development speed of the enzymatic cross-linking field is relatively slow. The enzymatic cross-linking is drawing attention as the next generation cross-linking technique for biomedical hydrogel fabrication, but unsolved limitations remain. In this section, we will briefly present current techniques in enzyme engineering, whose utility is widely proved or expected, and then consider how this can be applied to enzymatic cross-linking.

### A. Artificial enzymes

The advantages of the enzymatic cross-linking are the mild reaction conditions optimized for the physiological environment such as pH or temperature. However, we have also looked at examples where those advantages can sometimes be obstacles to stable cross-linking. In addition, hydrogels made by enzymatic cross-linking were often characterized by weak mechanical properties.^9^ To overcome this, several enzymes or chemical reagents have been mixed and used for cross-linking. On the other hand, artificial enzymes are actively researched in the field of protein engineering to improve the own performances of enzymes.^142–144^

**FIG. 10.**
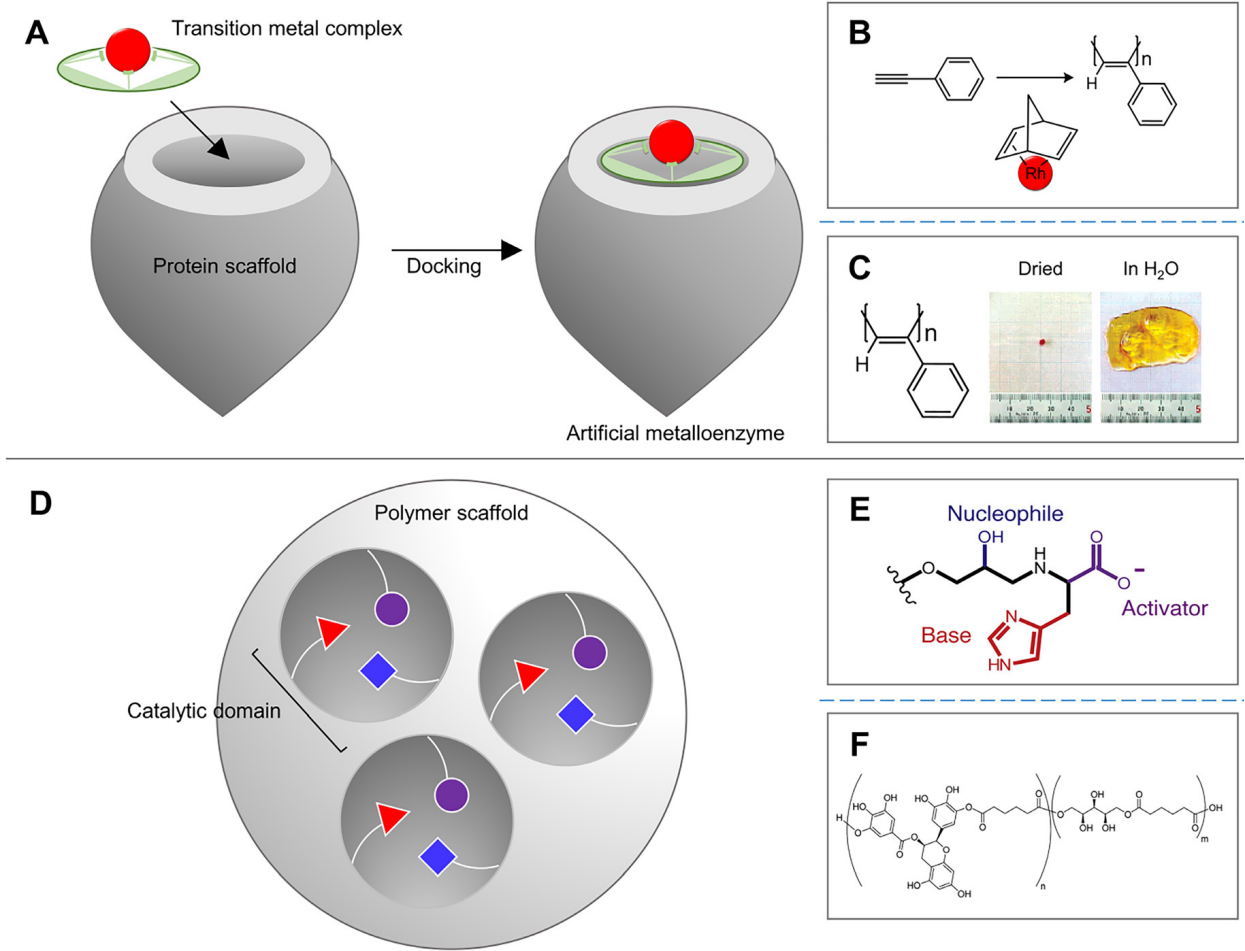
Artificial metalloenzyme and artificial scaffold-enzyme conjugates that mimic the catalytic domain of natural enzymes. (a) Schematic illustration of the artificial metalloenzyme. (b) Schematic illustration of rhodium catalyst-mediated polymerization of phenylacetylene. (c) Case of polyphenylacetylene hydrogel, which is fabricated by a rhodium catalyst. Reprinted with permission from Goto *et al.*, J. Am. Chem. Soc. **125**, 2516 (2003). Copyright 2003 American Chemical Society.^155^ (d) Schematic illustration of the nonmetal artificial enzyme, which consists of scaffold-enzyme conjugates. (e) Catalytic triad of artificial scaffold-conjugates mimicking catalytic triad of hydrolases—serine, histidine, and aspartate. Reprinted with permission from Nothling *et al.*, Chem **2**, 732 (2017). Copyright 2017 Elsevier.^158^ (f) Case of EGCG hydrogel, which is fabricated by a type of hydrolase—lipase. Reprinted with permission from Nitta *et al.*, J. Appl. Polym. Sci. **136**, 47693 (2019). Copyright 2019 Authors licensed under a Creative Commons Attribution (CC BY) license.^161^

Artificial enzymes are broadly divided into two types: artificial metalloenzymes (ArMs) [Fig. 10(a)] and nonmetal artificial enzymes [Fig. 10(d)]. ArMs mimic natural enzymes by combining the reactivity of the transition metal complex with the stability of the protein scaffold and have been studied relatively more than nonmetal artificial enzymes. The most often used ArM for enzymatic cross-linking is the artificial peroxidase, which can make hydrogels faster and in a more robust reaction compared to other enzymes. However, inflammation is also a problem to be solved.^145–149^ However, although research has been conducted separately within each research area, it is difficult to find a case that has been completed from the artificial enzyme development to its application to a hydrogel. Abe *et al.* combined a [Rh(nbd)Cl_2_] transition metal complex with apoferritin to perform phenylacetylene polymerization with catalytic activity superior to that of free [Rh(nbd)Cl_2_] [Fig. 10(b)].^150^ Onoda *et al.* conjugated the rhodium catalyst to the aponitrobindin scaffold to control the enantioselectivity of poly(phenylacetylene), a polymer of alkyne substituents.^151^ The polymer can create a helical structure by controlling the composition ratio of four conformational isomers (*cis*/*trans*-cis/transoid).^152,153^ Percec *et al.* used a free rhodium-norbornadiene catalyst to control the chirality of poly(phenylacetylene) by treating the solvent to change its helical structure,^154^ and Goto *et al.* added the chiral amine to more helical poly(phenylacetylene) [Fig. 10(c)].^155^ Connections between separated research areas are needed.

Although metalloenzymes have been preferred in the industry because of their stable and efficient reaction, biocompatible nonmetal enzymes attracted attention, especially in the field of bioengineering, because of the material contamination and toxicity issues of metalloenzymes.^156^ The main method to produce nonmetal artificial enzymes is to conjugate the natural-mimicking functional groups of the active site to the hydrophobic polymer/protein scaffold. Since only a very important part of the catalytic activity has been conjugated, it is possible to load several small units in one scaffold, and therefore, a more efficient reaction is achievable. For example, many hydrolases such as lipase B (CALB) from *Candida antarctica* and the esterase-mediate enzymatic ring-opening polymerization by Ser(-OH)-Hist(-Imidazole)-Asp(-CO_2_H) triad, which mimics this catalytic triad, have been used. Coulembier *et al.* showed the polymerization of L-lactic acid by implementing a catalytic triad of lipase with trifluoroacetate, imidazolium, and benzyl alcohol.^157^ Nothling *et al.* organized the hydrolytic functional groups into one functional unit and then loaded multiple units into one hydrophobic resin to maximize efficiency [Fig. 10(e)].^158^ Besides the aforementioned cases, there are many reactions mediated by ring-opening polymerization [Fig. 10(f)]^159–161^ and polymerization using the self-catalytic activity of proteins or nucleic acids.^162^ It can be observed in several cases that the artificial enzyme field and the hydrogel field are being researched separately, but if there is a connection point, it is expected that both fields will develop synergistically.

### B. Computational screening and modeling

Optimizing cross-linking conditions from the material to the enzyme for the hydrogel we would like to produce is complex, and finding the perfect enzyme is a very time-consuming and labor-intensive task. The directed evolution effectively reduces this complex process. However, the directed evolution also requires considerable effort due to system construction for mutagenesis and screening.^143^ In addition, the structure-functional characteristics of the new enzymes and the interaction with the substrate are so complex that the mechanism is not easy to study. Therefore, various computational methods are being researched and developed to reduce the labor load and increase the sophistication of research in a series of processes from directed evolution to mechanism modeling.

**FIG. 11.**
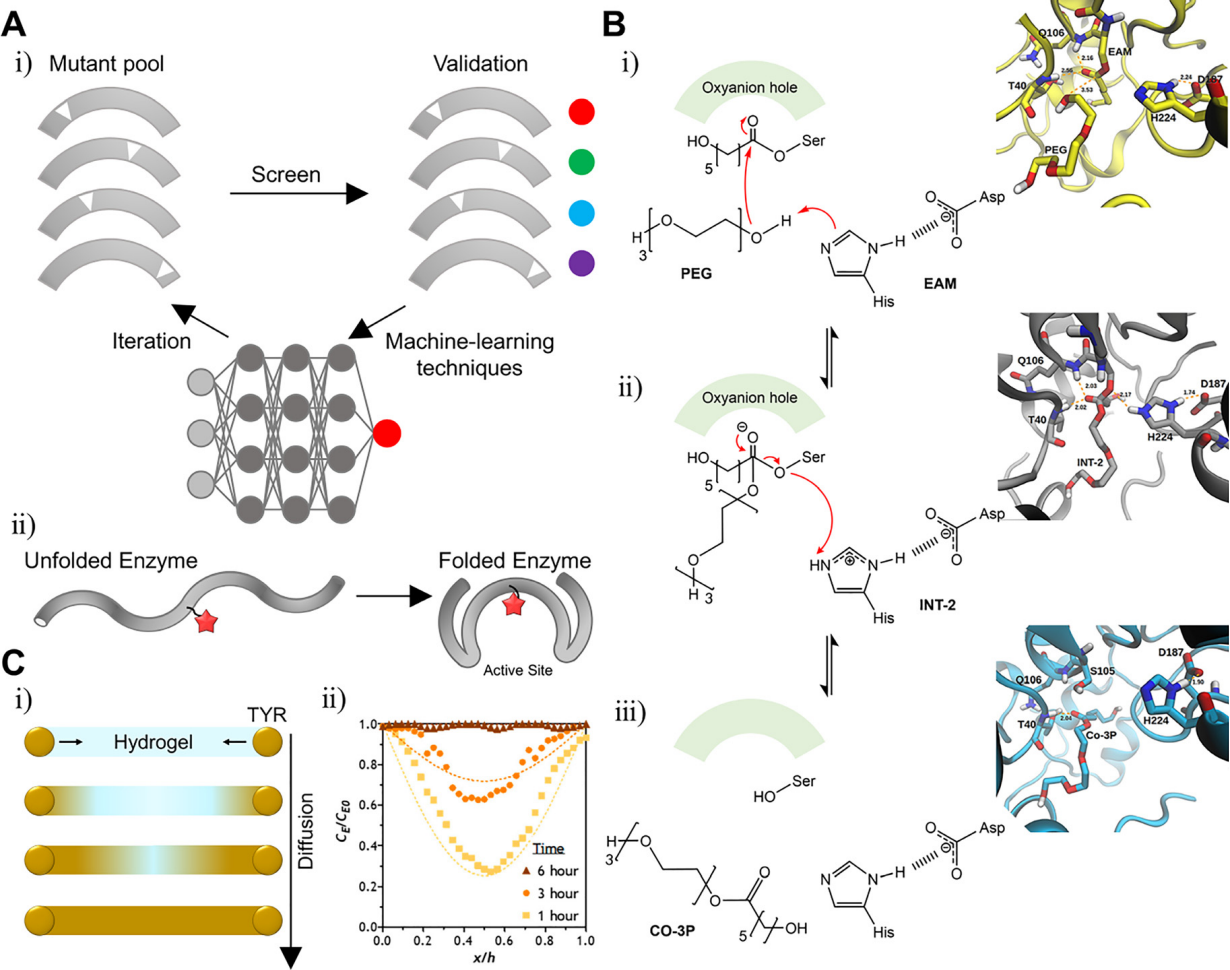
Computational screening and modeling techniques of enzyme engineering. (a) Schematic illustration of the enzyme screening process for directed evolution that is based on machine-learning techniques such as neural network (i). The ideal result of computation screening that predicts the stability of folding (ii). (b) QM/MM-based modeling of PCL-PEG copolymerization, which is mediated by lipase B from *Candida antarctica* (CaLB). Reprinted with permission from Figeiredo *et al.*, Front. Mol. Biosci. **6**, 109 (2019). Copyright 2019 Authors licensed under a Creative Commons Attribution (CC BY) license.^171^ (c) Computational modeling of gelation mediated by tyrosinase. Schematic illustration of tyrosinase diffusion into a PEG-peptide hydrogel during gelation (i) and comparison of the experimental value and simulational value (ii). Reprinted with permission from Liu *et al.*, Gels **5**, 1 (2019). Copyright 2019 Authors licensed under a Creative Commons Attribution (CC BY) license.^179^

First, for directed evolution, programs have been developed to simulate mutations based on the existing library and calculate their stability and functions. Upadhyay *et al.* developed a program called RisLNet to predict the stability of mutagenesis based on the existing protein library. In addition to the use of machine learning is a hot topic in the research area of directed evolution [Fig. 11(a-i)]. After learning the correlation between amino acid sequences and protein structures of existing protein data, the program begins to predict the structural and functional properties of the mutant [Fig. 11(a-ii)].^163^ Many researchers reported that predicting the mutant protein characteristics such as thermostability, ligand binding site prediction, enantioselectivity, and the structure was possible based on appropriate models appropriate for each condition.^164–169^ It is expected that the development of computational screening will promote the discovery of various new enzymes for cross-linking by innovatively saving labor and time.

Second, the computational modeling of the enzyme structure and kinetics has also been developed rapidly. For modeling, two major technologies are mainly applied, quantum mechanics (QM) and molecular mechanics (MM). In brief, QM calculates the electron/proton distribution interaction between each atom that composes the protein and the energy value of the reaction or transition state; based on the calculated value, the kinetics and stereospecificity of the enzyme can be interpreted. MM simulates the behavior of proteins based on the calculation of the potential energy for the intramolecular interaction of proteins. In contrast to QM, the creation and destruction of bonds cannot be simulated with MM. The QM/MM method, which complements the strengths and weaknesses of each, is commonly used. For example, the mechanism of rhodium catalyst-mediated phenylacetylene polymerization^170^ and PCL-PEG copolymerization^171^ was unveiled by the QM/MM method [Fig. 11(b-i-iii)]. Another modeling technology is, probably needless to say, machine learning. Since Google's AlphaFold showed an innovative neural network platform that can predict the 3D structure from protein amino acid sequence data by predicting the distance and binding angle of pairs of amino acid residues,^172^ various developments are based on their technologies.^173–176^ For example, the modeling of ε-caprolactone polymerization catalyzed by CALB has been reported.^177^ In other words, it is expected that research on the cross-linking mechanism of enzymes will be developed in the future to further enhance the understanding of the field of enzymatic cross-linking.

Besides the aforementioned modeling techniques focused on structural analysis, the modeling of the enzyme behavior in the hydrogel during cross-linking, such as diffusion, distribution, and activity maintenance in the hydrogel, is necessary, but research results are scarce. Even if the same dopamine is crosslinked, the tyrosinase reactivity varies greatly depending on whether the substrates are monomers or polymers, for example.^8^ Interestingly, computational modeling of the enzyme dynamics during the cross-linking environment has recently been reported. Valero *et al.* modeled the cross-linking of collagen hydrogel using transglutaminase through the worm-like chain (WLC) method,^178^ and Liu *et al.* modeled how tyrosinase diffuses and maintains its catalytic activity in the hydrogel environment [Fig. 11(c-i-ii)].^179^

Enzyme screening and modeling techniques based on computational analysis are under development and applied to the fields of enzymatic cross-linking. Following this trend, it is expected that more high-performance enzymes will be developed and innovative material production systems will be prepared.

### C. Engineered living materials

Materials produced by the living system's metabolic pathways or genetically modified mechanisms are called engineered living materials (ELMs). The research area of ELMs is an emerging field due to the characteristics of living things such as evolvability, self-organization, and responsiveness to the environment.^180^ Therefore, although there are a few cases reported, we will briefly describe micro-organism-based ELMs and multicellular living organism-based ELMs.

**FIG. 12.**
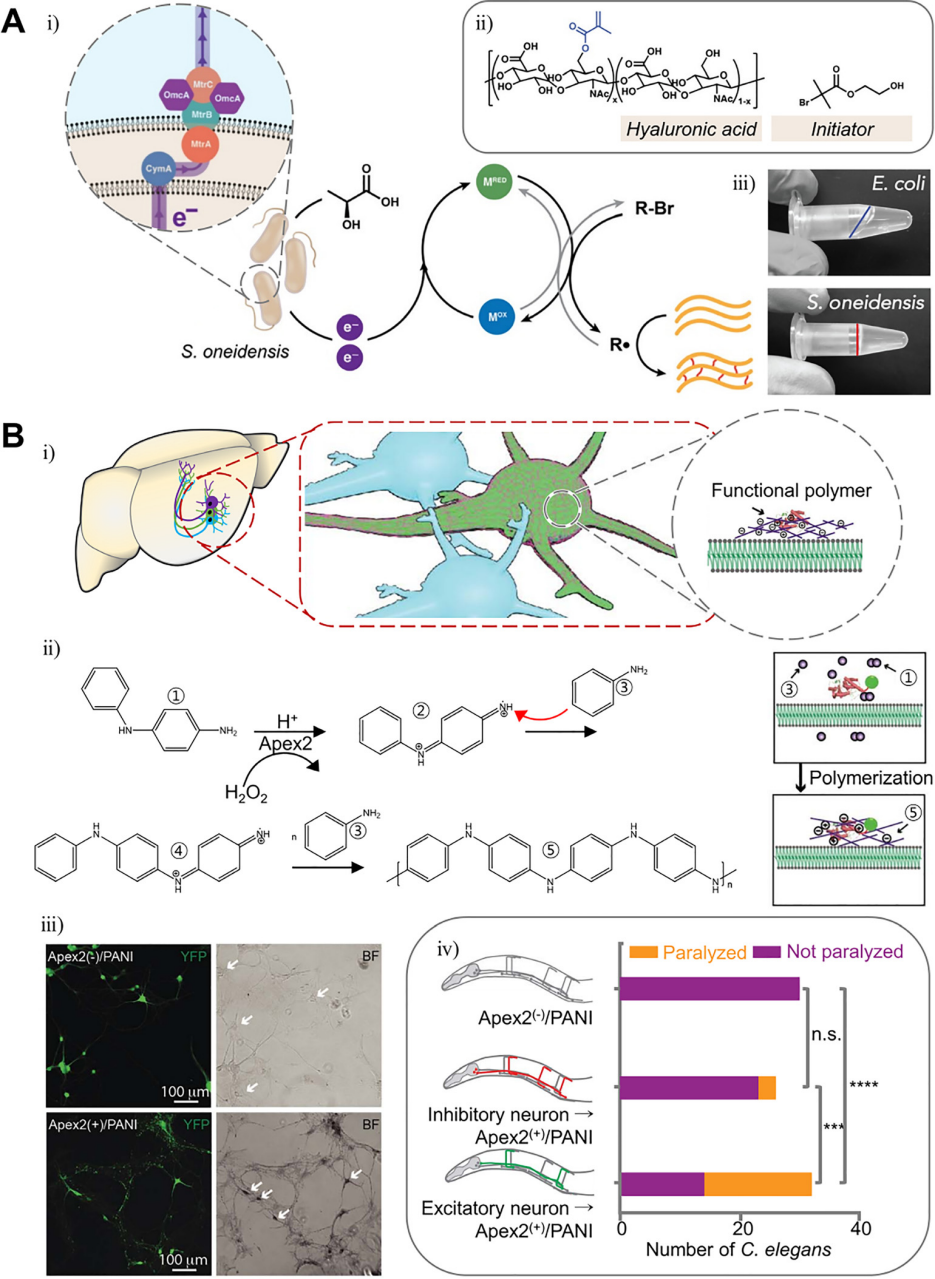
Polymer synthesis of living organisms by genetically engineered enzymes. (a) Schematic illustration of methacrylated hyaluronan hydrogel fabrication by extracellular electron transfer (EET)-controlled polymerization (i). Crosslinking components of the gel (ii). Polymerization product (iii). Reprinted with permission from Graham *et al.*, ACS Biomater. Sci. Eng. **6**, 1375 (2020). Copyright 2020 American Chemical Society.^183^ (b) Schematic illustration of neuronal membrane-specific polymerization of aniline by genetically targeted chemical assembly (i). Schematic illustration of aniline polymerization mediated by Apex2 (ii). The polyaniline on neuron cells of rat hippocampus (iii). Behavioral analysis of *C. elegans*, comparing the effect of polymerization between different neuronal types. Reprinted with permission from Kim *et al.*, Science **367**, 1372 (2020); Copyright 2020 The American Association for the Advancement of Science.^184^

The bacterial metabolism has been widely used in all aspects of human life, including industry, agriculture, and medicine.^181^ Therefore, it is not surprising that bacterial enzymes are also used for materials. As genome editing becomes more convenient with the development of the CRISPR-Cas9 system, investigations on the production of recombinant polymers through bacterial metabolism have more been actively reported. As a result, polymers such as polyhydroxyalkanoates (PHA), hyaluronate (HA), and poly (γ-d-glutamic acid) (γ-PGA) have already been mass-produced industrially.^182^ Further on, research into the hydrogel fabrication through bacterial metabolism has been recently reported [Fig. 12(a-i)]. Graham *et al.* showed the hydrogel formation of methacrylate-functionalized hyaluronate (MeHA) and radical initiator 2-hydroxyethyl 2-bromoisobutyrate (HEBIB) by radical cross-linking using extracellular electron transfer (EET)-controlled ATRP of *Shewanella oneidensis* [Fig. 12(a-ii-iii)].^183^ Crosslinking may be adjusted by knocking out the EET-associated genes of *S. oneidensis*. MtrC, a key electron transfer protein, can also be regulated under transcriptional control.

ELMs based on multicellular biological systems are not yet actively researched, but Liu *et al.* recently reported an innovative enzymatic polymerization method for the metazoan nervous system using genetically targeted chemical assembly (GTCA) [Fig. 12(b-i)].^184^ The rat hippocampus and *Caenorhabditis elegans* (*C. elegans*) were transduced humanized ascorbate peroxidase (APEX2) and normal Apex2, respectively, followed by perfusion of aniline or 3,3′-diaminobenzidine into model organisms [Fig. 12(b-ii)]. Through oxidative radical polymerization, polyaniline (PANI) or poly(3,3′-diaminobenzidine) (PDAB) is specifically deposited only on APEX2(Apex2)-expressing neurons [Fig. 12(b-iii)]. Neurons with PANI or PDAB deposition were able to identify the increase in capacitance due to the polymer conductive property while maintaining viability. Interestingly, *C. elegans* behaviors were changed depending on which type of neuron was targeted (cholinergic excitatory motor neuron or GABAergic inhibitory motor neuron) [Fig. 12(b-iv)]. When excitatory neurons were targeted, *C. elegans* was paralyzed. This study is an innovation of mixing enzyme engineering and polymer chemistry. We expect that new hydrogels using the structural and functional complexity of the multicellular biological system will be developed through various enzymatic cross-linking methods.

## VI. CONCLUSION AND FUTURE PERSPECTIVES

Enzymatic cross-linking, which occurs in biological systems for ECM synthesis, may also be applied for hydrogel fabrication. Mainly, oxidoreductase (tyrosinase and HRP) or acyl-transferase (transglutaminase or sortase A) has been used for enzymatic cross-linking and robust chemical cross-linking. Reactions mediated by such enzymes are advantageous for biomedical applications due to enzyme specificity and biocompatibility. The applicability to various platforms such as 3D printing, tissue adhesion, injection, and spraying is also advantageous for biomedical applications.

For higher functionality of enzymatic cross-linking, screening enzymes whose cross-linking activities are favorable under physiological conditions have been actively researched. Besides, innovative engineering techniques such as directed evolution and protein modification are progressing fast, affected by various modern computational high-throughput techniques such as machine learning. Still, there are some problems to be solved in the research area of enzymatic cross-linking, such as the immunogenicity of HRP or the mechanism modeling complex reactions mediated by multiple enzymes. However, the progress in protein engineering and increasing attention in the enzymatic based cross-linking system would allow the fabrication of novel hydrogel materials for biomedical applications.

## AUTHORS' CONTRIBUTIONS

W.S. and J.K. contributed equally to this work.

## Data Availability

Data sharing is not applicable to this article as no new data were created or analyzed in this study.
